# Discovery and characterization of *Christensenella hongkongensis* as a novel bacterium in the adenoma-carcinoma progression

**DOI:** 10.1186/s12967-026-07886-9

**Published:** 2026-02-28

**Authors:** Wenqing Zhang, Qi Su, Haiyun Shi, Yang Sun, Xiaobo Li, Mengbin Li, Hui Wang, Jun Yu, Nathalie Wong, Francis Ka Leung Chan, Jingwan Zhang, Siew Chien Ng

**Affiliations:** 1 Microbiota I-Center, Hong Kong SAR, China; 2 Department of Medicine and Therapeutics, The Chinese University of Hong Kong, Hong Kong SAR, China; 3 Beijing Friendship Hospital, Capital Medical University, Beijing, China; 4 The First Affiliated Hospital of Kunming Medical University, Kunming, Yunnan China; 5 Renji Hospital, Shanghai Jiao Tong University School of Medicine, Shanghai, China; 6 Xijing Hospital, Xi’an, Shaanxi China; 7 The Sixth Affiliated Hospital, Sun Yat-Sen University, Guangzhou, Guangdong, China; 8 Li Ka Shing Institute of Health Sciences, State Key Laboratory of Digestive Disease, Institute of Digestive Disease, The Chinese University of Hong Kong, Hong Kong SAR, China; 9 Department of Surgery, Sir Y.K. Pao Centre for Cancer, The Chinese University of Hong Kong, Hong Kong SAR, China; 10 Centre for Gut Microbiota Research, The Chinese University of Hong Kong, Hong Kong SAR, China; 11 New Cornerstone Science Laboratory, The Chinese University of Hong Kong, Hong Kong SAR, China

**Keywords:** Colorectal adenoma, Biomarker, Gut microbiota, *Christensenella hongkongensis*

## Abstract

**Background:**

Colorectal cancer (CRC) is one of the most prevalent malignancies worldwide and commonly starts from a pre-cancerous stage. This study aimed to identify potential fecal bacterial candidates associated with progression of CRC from the adenoma-carcinoma sequence and to explore underlying mechanisms of carcinogenesis.

**Methods:**

Publicly metagenomic datasets were analyzed using MaAsLin2 to identify bacterial species enriched in CRC patients compared to healthy controls. Additionally, we established a large cohort in mainland China, consisting of 686 subjects, including 285 CRC patients, 73 advanced adenoma patients (AA), 134 non-advanced adenoma patients (nAA), and 194 healthy controls (NC). Fecal samples from this cohort were analyzed by duplex quantitative polymerase chain reaction (qPCR) to validate the abundance of key bacterial candidate and its association with tumor node metastasis (TNM) stages. Receiver operating characteristic (ROC) curve analysis was performed to evaluate the diagnostic performance of *Christensenella hongkongensis* (*C. hongkongensis*) alone and in combination with fecal immunochemical test (FIT) across different CRC stages. In vitro experiments and transcriptome sequencing were performed to explore the effects of *C. hongkongensis* and its mechanisms in CRC progression.

**Results:**

MaAsLin2 analysis identified seven bacterial species were significantly more abundant in fecal samples of CRC patients than in healthy controls (*p* < 0.05). Among them, *C. hongkongensis*, an obligately anaerobic, catalase-positive, motile, non-sporulating, gram-positive coccobacillus was distinguished by its lowest abundance in healthy controls and significant enrichment in CRC patients. Validation in our recruited cohort showed that the abundance of *C. hongkongensis* progressively increased from non-advanced adenomas to advanced adenomas and CRC. For classifying AA from nAA, *C. hongkongensis* yielded an area under the ROC curve (AUC) of 0.60 (95% CI 0.53–0.68), with 45.2% sensitivity and 85.8% specificity. A combined model integrating *C. hongkongensis* abundance and FIT further improved diagnostic performance, increasing AUCs from 0.77 to 0.81 for AA vs NC (*p* < 0.05) and from 0.76 to 0.82 for AA vs nAA (*p* < 0.001). Linear regression analysis revealed a significant positive association between *C. hongkongensis* and TNM stages in CRC. In vitro experiments showed that *C. hongkongensis* promoted CRC cell proliferation, inhibited apoptosis, and enhanced the growth of patient-derived CRC organoids. RNA-seq analysis identified activation of the Wnt/β-catenin signaling pathway, which was further validated by elevated protein levels of active β-catenin, reduced phosphorylation of GSK3β, and the upregulation of downstream targets *c-Jun* and *Cyclin-D1*.

**Conclusions:**

Our findings suggest that *C. hongkongensis* promotes colorectal tumorigenesis *via* Wnt/β-catenin activation, and highlight its potential as a novel non-invasive bacterial marker for early detection and monitoring of CRC progression.

**Graphical Abstract:**

**Supplementary Information:**

The online version contains supplementary material available at 10.1186/s12967-026-07886-9.

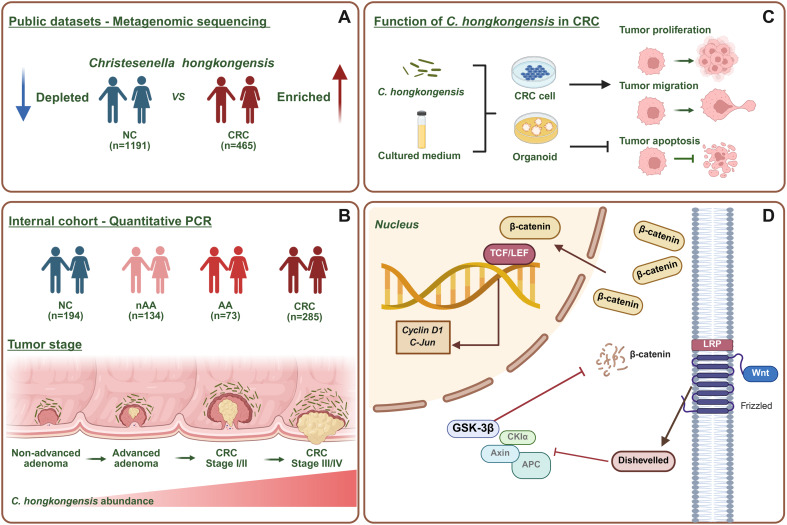

## Introduction

Colorectal cancer (CRC) is the third most common malignancy and the second leading cause of cancer-related mortality worldwide, with its incidence continuing to rise and imposing substantial medical and economic burdens [1, 2]. Most cases of CRC originate from precursor lesions, including non-advanced adenomas, advanced adenomas, and serrated polyps, each carrying distinct risks of progression to cancer [3]. Among these, advanced adenomas present a significantly higher risk of developing into CRC compared to non-advanced adenomas [4]. Early detection and endoscopic removal of precursor lesions are effective strategies for markedly reducing CRC risk [5]. Although the fecal immunochemical test (FIT) is the most widely used non-invasive method for early CRC detection, its sensitivity for identifying advanced adenomas remains suboptimal, ranging from 25 to 42% [3]. Consequently, there is an urgent need for novel biomarkers with greater accuracy to improve the detection of precancerous lesions and further reduce the incidence and mortality of CRC [6, 7].

Accumulating evidence, including findings from animal studies, has linked dysregulation of the gut microbiota to the progression of CRC, which is characterized by reduced microbial diversity and expansion of pathobiont [8]. Microbial depletion, achieved either through germ-free conditions [9] or antibiotic intervention [10], significantly reduces colorectal tumor formation compared to conventional microbiota. Pathogenic bacteria such as *Fusobacterium nucleatum* (*F. nucleatum*) [11]*, Peptostreptococcus stomatis* [12], *pks+ Escherichia coli* [13], and *Enterotoxigenic Bacteroides fragilis* [14] are enriched in CRC patients relative to healthy controls. These pathobionts contribute to CRC initiation and progression through multiple mechanisms, including the production of carcinogenic genotoxins and tumorigenic metabolites, bacterial adhesin-host cell receptor interactions, genetic and epigenetic modifications, and the induction of inflammation [8]. These findings underscore the crucial role of the gut microbiota in CRC pathogenesis and highlights its potential as a potential biomarker for non-invasive CRC diagnosis.

Stool-based bacterial markers have been shown to serve as non-invasive diagnostic tools for early CRC detection *via* targeted quantification using quantitative polymerase chain reaction (PCR) [15]. For example, *F. nucleatum* and the novel gene marker “m3” from *Lachnoclostrium sp*. are significantly enriched during the progression from colorectal adenoma to CRC, showing good diagnostic performance for CRC [5]. However, their accuracy for adenoma diagnosis decreases significantly to 48%, and “m3” does not significantly distinguish between non-advanced and advanced adenomas [5]. These findings highlight the current limitations on biomarkers discovery for effective colorectal adenoma detection.

*Christensenella hongkongensis* is an anaerobic, gram-positive bacterium that forms a distinct branch within the *Christensenella* genus and carries several antibiotic resistance genes [16, 17]. In contrast, other members of the *Christensenella* genus, such as *Christensenella minuta* [18], which have been reported to exhibit probiotic properties including anti-inflammatory effects in inflammatory bowel disease, recent studies have revealed that *C. hongkongensis* is enriched in CRC patients and is associated with bacteremia; however, its precise role in CRC remains unclear.

Hence, this study aims to evaluate potential of *C. hongkongensis* as a bacterial marker for early CRC detection through metagenomic sequencing analysis and qPCR validation, and to explore its molecular mechanisms in promoting CRC development *via in vitro* experiments. The findings of this study are expected to contribute to the development of new diagnostic tool for tracking the adenoma-to-carcinoma progression in colorectal cancer and provide valuable insights for its prevention and treatment.

## Materials and methods

### Data download and metagenomic analysis

A total of 1656 fecal metagenomic sequencing samples from colorectal cancer patients and healthy controls, were collected from three datasets conducted in Hong Kong and Japan. The Hong Kong cohort (174 CRC, 893 controls) derived from our previous study was used as the discovery cohort. Two public cohorts from Japan were designated as validation cohort 1 (40 CRC, 40 controls) and validation cohort 2 (251 CRC, 258 controls). Bacterial taxonomic profiles were generated using MetaPhlAn (v0.3.0.13) [19] and accessed by the *curated Metagenomic Data* package (v0.3.14.0) [20] in R. Differential species between CRC patients and controls were identified by MaAslin2 (v0.1.20.0) across all cohorts. The abundance of *C. hongkongensis* was specifically compared using Mann-Whitney test in each cohort. The characteristics of all datasets are provided in Supplementary Table 1.

### Human subject recruitment

Participants were included if they were between 40 and 75 years old and had undergone a standard colonoscopy. Participants were excluded from the study if they had active gastrointestinal or had used antibiotics within the past month. Healthy controls were defined as individuals with normal colorectal mucosa and no evidence of gastrointestinal disorders, including IBD, irritable bowel syndrome (IBS), colorectal adenoma and CRC, as confirmed by colonoscopy examination showing no detectable lesions or abnormalities. Subjects with colorectal adenoma and CRC were diagnosed through colonoscopy and confirmed by histological examination. The TNM stage for CRC patients was assessed by the AJCC (American Joint Committee on Cancer) TNM system.

Finally, we recruited 686 consecutive subjects, including 285 CRC, 73 advanced adenomas, 133 non-advanced adenomas and 194 healthy controls from five different sites (Guangzhou, Beijing, Shanghai, Kunming and Xi’an) of mainland China. Advanced adenomas (AA) were defined as adenomas with any of the following criteria: size > 10 mm, more than three adenomas, the presence of a tubulovillous or villous component, or high-grade intraepithelial dysplasia. Non-advanced adenomas were defined as 1–2 adenomas, each < 10 mm in size [21]. Tumor anatomic locations were typically divided into three categories: rectum, proximal colon, and distal colon. The rectum refers to the terminal segment of the larger intestine. The proximal colon includes the ileocecal junction, ascending colon, hepatic flexure and transverse colon. The distal colon includes the splenic flexure, descending colon and sigmoid colon. Clinical characteristics of healthy subjects and colorectal adenoma/cancer patients are shown in Supplementary Table 2.

### Fecal sample collection

Fecal samples were collected within one month prior to the scheduled colonoscopy and before any bowel preparation. The fresh fecal sample was collected from each participant using a sealed, sterile collection tube containing preservative media (Norgen Biotek Corp, Thorold, Ontario Canada). All samples were kept capped when not in use to prevent cross-contamination. Following collection, samples were sent to the lab within 24 h and stored at −80 °C until analysis.

### Fecal DNA extraction

For mainland China cohort, fecal DNA was extracted following manufacturer’s instruction of BayBiopure Magnetic Stool Nucleic Acid Kit (Baybio, Guangzhou, China). The extracted DNA was quantified by NanoDrop 2000 spectrophotometer (Thermo Fisher Scientific, Waltham, MA, USA). The average DNA yield was 499.19 ± 11.25 ng/μL, with A260/280 ratios ranging from 1.7 to 2.2 and A260/A230 ratios ranging from 1.8 to 2.2, indicating acceptable DNA purity. After final precipitation, the DNA samples were resuspended in DE buffer and stored at −80 °C before further analysis. The DNA concentration of each subject are shown in the Supplementary Table 3.

### Duplex quantitative PCR

Duplex quantitative PCR (qPCR) was performed using the Applied Biosystems™ 7500 system to quantify the abundance of *C. hongkongensis* in fecal samples from mainland China cohort. Each 20 μL reaction contained 10.2 μL Premix Ex Taq™ (TAKARA, Tokyo, Japan), 0.4 μL each of *C. hongkongensis* forward and reverse primers (10 μM), 0.2 μL *C. hongkongensis* probe labeled with FAM (10 μM), 0.4 μL each of 16S forward and reverse primers (10 μM), 0.2 μL 16S probe labeled with VIC (10 μM), 5.8 μL nuclease-free water, and 2.0 μL template DNA (5 ng/μL). The qPCR program consisted of a pre-denaturation step at 95 °C for 30s, followed by 40 cycles of denaturation at 95 °C for 5s and annealing/extension at 60 °C for 34s. The amplification efficiency of the *C. hongkongensis* and 16S rRNA primer sets was validated by generating standard curves using 10-fold serial dilutions of template DNA (Fig. S2), showing efficiencies of 90.25% and 91.29%, respectively, with a correlation coefficient (R^2^ = 0.99). Primer specificity was confirmed by melting curve analysis. The relative abundance of *C. hongkongensis* was determined by the 2^−^ ΔΔCt method, with the 16S rRNA gene as an internal reference. Ct values were analyzed with a threshold of 0.07 and a baseline set between cycles 3 and 15. The sequences of the gene primers are provided in Supplementary Table 4.

### Fecal immunochemical test

Fecal occult blood was detected by fluorescence immunochromatographic fecal immunochemical test. All procedures were performed in accordance with the manufacturer’s instruction. Stool samples were collected with a fecal sampling tube and thaw on the ice. The sampling stick was inserted into several parts of the stool until the grooves were filled, then returned to the tube and capped tightly. The samples were mixed thoroughly by vortexing for 30 s and centrifuged at 3500 rpm for 5 mins. The supernatant was used immediately or stored at 4 °C for up to 12 h. After equilibration of the reagent kit at 10–30 °C, 100 μL of supernatant was added to the sample well of the test card. After sample loading for 15 mins, the test card was inserted into the DFIA300 analyzer (Guangzhou Bio-Creat Biotechnology Co., Ltd., China) for measurement. The instrument automatically calculated the fluorescence intensity and converted it to hemoglobin concentration (μg/g). A result above the manufacturer’s cutoff value (34 μg/g) was considered FIT-positive, indicating the presence of fecal hemoglobin. The detailed hemoglobin concentration and FIT results are provided in Supplementary Table 3.

### Receiver operating characteristic (ROC) curve analysis

ROC curve analyses were performed using pROC package (v1.18.2) in R. For each pairwise classification (CRC vs nAA, CRC vs NC, AA vs nAA, and AA vs NC), the diagnostic performance of *C. hongkongensis* (Chk) and FIT were assessed individually and in combination. The FIT result was treated as a dichotomous variable (positive = 1, negative = 0), whereas the relative abundance of *C. hongkongensis* was treated as a continuous variable. The combined diagnostic model was constructed by binary logistic regression, using disease status (case = 1, control = 0) as the dependent variable and both *C. hongkongensis* abundance and FIT as independent predicators. Predicted probabilities from these models were used as combined predictors for ROC analysis. Area under the ROC curve (AUC) values and 95% confidence intervals (95% CI) were estimated by a non-parametric method, and Delong’s test was used for pairwise comparisons of ROC curves. The best cutoff values for each model were determined by maximized the Youden index (J = Sensitivity + Specificity − 1). At the optimal cut-off point, the sensitivity and specificity were calculated to evaluate diagnostic performance.

### Bacteria culture

*C. hongkongensis* (DSM18959) was purchased from Leibniz Institute DSMZ-Germany Collection of Microorganisms and Cell Cultures GmbH and cultured at 37 °C in Peptone-Yeast Extract-Glucose (PYG) broth with Hungate bottles filled with nitrogen. A nonpathogenic human commensal intestinal reference strain [22], *Escherichia coli* MG1665, which has been reported not to promote colorectal carcinogenesis [23], was used as bacterial control and cultured at the same condition as *C. hongkongensis*. Furthermore, *E. coli* MG1655 is commonly used as a negative control in animal models of various diseases [24, 25]. When the absorbance of *C. hongkongensis* reached optical density 600 of 0.3, the conditioned medium was centrifuged and filtered with 0.22 μm pore size filter to collect *C. hongkongensis* conditioned medium (Chk.CM) or *E. coli* conditioned medium (E. coli.CM). The multiplicity of infection (MOI) = 100 was selected based on previous studies [26–28] investigating host-bacteria interactions in colorectal cancer models in vitro, where this ratio has been widely applied to examine bacterial effects on epithelial and tumor cells. This MOI offers a reproducible infection condition that maintains cell viability while eliciting measurable biological responses. We observed that 5% conditioned medium did not alter CRC cell line or organoid viability, whereas higher concentrations (10%–15%) caused organoid burst. Therefore, 5% was chosen as an optimal concentration to evaluate the metabolic impact of *C. hongkongensis* without inducing cytotoxic effects.

### Cell culture

Normal human colon epithelial cell NCM460, human CRC cell lines HCT116 and CaCo-2 were obtained from ATCC (Rockville, MD, USA). All cell lines were cultured in DMEM (Thermo Fisher Scientific) supplemented with 10% fetal bovine serum (FBS) and 1% penicillin-streptomycin (P/S) in humidified atmosphere containing 5% CO_2_.

### Human CRC organoid culture

The basic medium is Advanced DMEM/F12 (Gibco), supplemented with 1X P/S, 10 mM HEPES, 1X GlutaMAX medium containing 1X N2 and B27 supplements, 100 ng/mL R-spondin-1, 100 ng/mL Noggin, 50 ng/mL EGF, 10 nM Gastrin, 100 ng/mL FGF10, 1.25 mM N-acetylcysteine, 500 nM A8301, 10 μM SB202190, 10 mM Nicotinamide, 100 μg/mL Primocin and 10 μM Y27632.

A CRC patient-derived organoid line (passage 6) was obtained from Prof Nathalie Wong’s laboratory, isolating from a 75-year-old male colorectal adenocarcinoma patient. CRC organoids were activated and embedded into Matrigel (Corning), and seeded into pre-warmed 24-well plates, followed by incubation at 37 °C and 5% CO_2_ for 30 mins to allow Matrigel polymerization. The organoids were then maintained in CRC organoid culture medium at 37 °C with 5% CO_2_ for 2–3 weeks.

To passage organoids from Matrigel, gentle pipetting with cold PBS was employed. Mechanical shearing and digestion using TryplE Express (Thermo Fisher Scientific, USA) was used to break organoids into single cells or small fragments. Following centrifugation, organoid fragments were used for passaging, cryopreservation, or other experimental procedures.

Organoids at passages 7–9 were used for all experiments. Treatment such as 5% (*vol/vol*) PYG or Chk.CM was added into the culture medium directly, which was freshly changed every three days. Surface area of organoid was measured by Image J 1.54 g.

### Cell proliferation assay

CaCo-2, HCT116 and NCM460 cells (1 × 10^3^ cells per well) were seeded onto 96-well plates and treated with live *E. coli* (CFU = 10^6^/mL) or *C. hongkongensis* (CFU = 10^6^/mL), as well as 5% (*vol/vol*) PYG, E. coli.CM, or Chk.CM in DMEM. Cell viability was determined by Cell Counting Kit-8 (CCK8, MedChemEXpress, Monmouth Junction, NJ, USA; Hy-K0301) for 3 consecutive days.

### Cell apoptosis assay

CaCo-2, HCT116 and NCM460 cells (5 × 10^5^ cells per well) were separately seeded in a 12-well plate and incubated until reaching approximately 80–90% confluence. Cells were infected with *C. hongkongensis* (MOI = 100) for 4 hours under anaerobic conditions. Following infection, cells were washed 2–3 times with 1X PBS and replaced with DMEM supplemented with antibiotics to prevent further bacterial growth. After 48 hours post-infection, cells were washed twice with cold 1X PBS and detached by adding 0.5 mL trypsin without EDTA to each well. The cell suspension was collected and centrifuged at 1000 rpm for 5 mins at 4 °C. The resulting cell pellet was resuspended in 1X Annexin V bindng buffer to achieve a final concentration of 1 × 10^6^ cells/mL. A 100 µL aliquot of the resuspended cells (1 × 10^5^ cells) was transferred to a 5 mL flow cytometry tube. Cells were stained using the BD Pharmingen™ PE Annexin V Apoptosis Detection Kit I (BD Biosciences, San Jose, CA, USA) according to the manufacturer’s protocol. After staining, 400 µL of 1X binding buffer was added to each tube, and samples were analyzed immediately by flow cytometry.

### Wound healing assay

CaCo-2 and HCT116 cells (1 × 10^3^ cells per well) were seeded in 6-well plates and cultured until confluence. A uniform scratch was carefully made in the cell monolayer using a sterile 200 µL pipette tip, followed by washing with PBS to remove debris. The cells were then incubated in serum-free medium under the following conditions [1] live *C. hongkongensis* (MOI = 100) [2], serum-free medium (blank control for *C. hongkongensis*) [3], 5% Chk.CM, and [4] 5% PYG medium (negative control for Chk.CM). Images of the scratch areas were captured at 0 h and 12 h, and scratch widths were measured using ImageJ software to assess wound closure.

### Colony formation assay

HCT116 and CaCo-2 cells (1 × 10^3^ cells per well) were seeded in 6-well plates. For live *C. hongkongensis* treatment, cells were co-cultured with live *C. hongkongensis* (MOI = 100) for 4 hours. Live *E. coli* MG1655 (MOI = 100) served as a negative control. After 4 hours, cells were washed three times with PBS and fresh DMEM medium was added. For *C. hongkongensis* treatment, cells were cultured with 5% Chk.CM, while 5% PYG medium and E. coli.CM were used as blank and negative controls, respectively. The treatment medium was refreshed every 3 days. After 10–14 days, cells from both treatments were fixed with 4% PFA and stained with 0.1% crystal violet. Colonies containing more than 50 cells were counted. All experiments were performed in triplicate and repeated three times.

#### RNA sequencing analysis

Total RNAs were extracted from the HCT116 and CaCo-2 cells after co-culturing with live *C. hongkongensis* (MOI = 100) or 5% Chk.CM respectively using Trizol Reagent (Thermo Fisher Scientific). RNA sequencing libraries were prepared with at least 6 G raw data per sample (NovaSeq PE150, Novogene, Beijing, China). Raw sequencing data were qualified and cleaned using fastp (v0.0.24.0) [29] and TrimGalore (v0.0.6.10). The processed reads were then aligned to the reference human genome (GRCh38) using3Hisat2 (v0.2.1.0–4) [30]. Differential gene expression analysis was performed with DESeq2 (v0.1.46.0) [31], applying the thresholds of |Log2FoldChange|>1 and adjusted *P*-value < 0.05 to identify significantly differential genes. The identified differentially expressed genes were subjected to KEGG enrichment analysis using the ClusterProfiler (v0.4.14.6) [32]. Additionally, Gene Set Enrichment Analysis (GSEA) was conducted *via* the GSEA software [33] with default parameters using the KEGG pathway database. Finally, pathway visualizations of differential gene expression were generated using the Pathview (v0.1.46.0) [34].

### RT-PCR

cDNA was synthesized from total RNA by PrimeScript RT reagent Kit (RR037A, Takara, Tokyo, Japan) according to the manufacturer’s instructions. Quantitative PCR (qPCR) was conducted using TB Green® Premix Ex Taq™ II (TAKARA) on the QuantStudio 7 Flex System (Thermo Fisher Scientific). The sequences of the primers are listed in Supplementary Table 4.

### Western blot

The total proteins were isolated from cells or organoid samples using IP lysis buffer (Thermo Fisher Scientific) supplemented with PMSF and protease and phosphatase inhibitor cocktail (MCE, Shanghai, China). Protein concentrations were measured by BCA protein assay kit (Thermo Fisher Scientific); Equal amounts of protein (50 µg per sample) were separated on 10% SDS-PAGE gels and then transferred onto 0.45 µm PVDF membranes. After blocking with 5% BSA, blots were incubated with the primary antibodies listed in Supplementary Table 5 overnight at 4 °C and secondary antibodies for 1 h at room temperature, respectively. Membranes were exposed to clarity Western ECL substrate (Bio-Rad; California, USA). Band intensities were determined using ImageJ 1.54 g (National Institutes of Health).

### Statistical analysis

For *C. hongkongensis* abundance, which is expressed as mean±SE or median (interquartile, IQR) as appropriate. For single-cohort comparisons based on metagenomic data, nonparametric Mann–Whitney U tests were used to assess statistical significance between CRC and control groups. For the multicenter qPCR cohort from mainland China, differences in the fecal relative abundance of *C. hongkongensis* among clinical groups were evaluated using linear mixed-effects models fitted with the lmer function in the lme4 (v0.1,1.37) R package, with clinical group as a fixed effect and study center as a random intercept. One-way ANOVA with multiple comparisons and a test for linear trend was used to evaluate changes of C. *hongkongensis* levels during disease progression (from nAA and AA to CRC; and across TNM stages). Differences in the prevalence of C. *hongkongensis* among groups were analyzed using the χ^2^ test. The diagnostic performance of *C. hongkongensis* was analyzed by calculating the area under the receiver-operating characteristic (ROC) curve (AUC) by pROC (v0.1.18.2) package. The experimental data are generally represented as mean±SD. Paired Student’s t-test and one-way ANOVA with Tukey’s multiple comparison test were used to evaluate the statistical significance of differences between two groups and among three groups, respectively. The graphical abstract was created with BioRender.com. GraphPad Prism 10 Software and SPSS version 27.0 software were used for data analysis in this study. *p* value < 0.05 was considered as statistical significance.

## Results

### Identification of the novel species *C. hongkongensis* enriched in patients with CRC

**Fig. 1:**
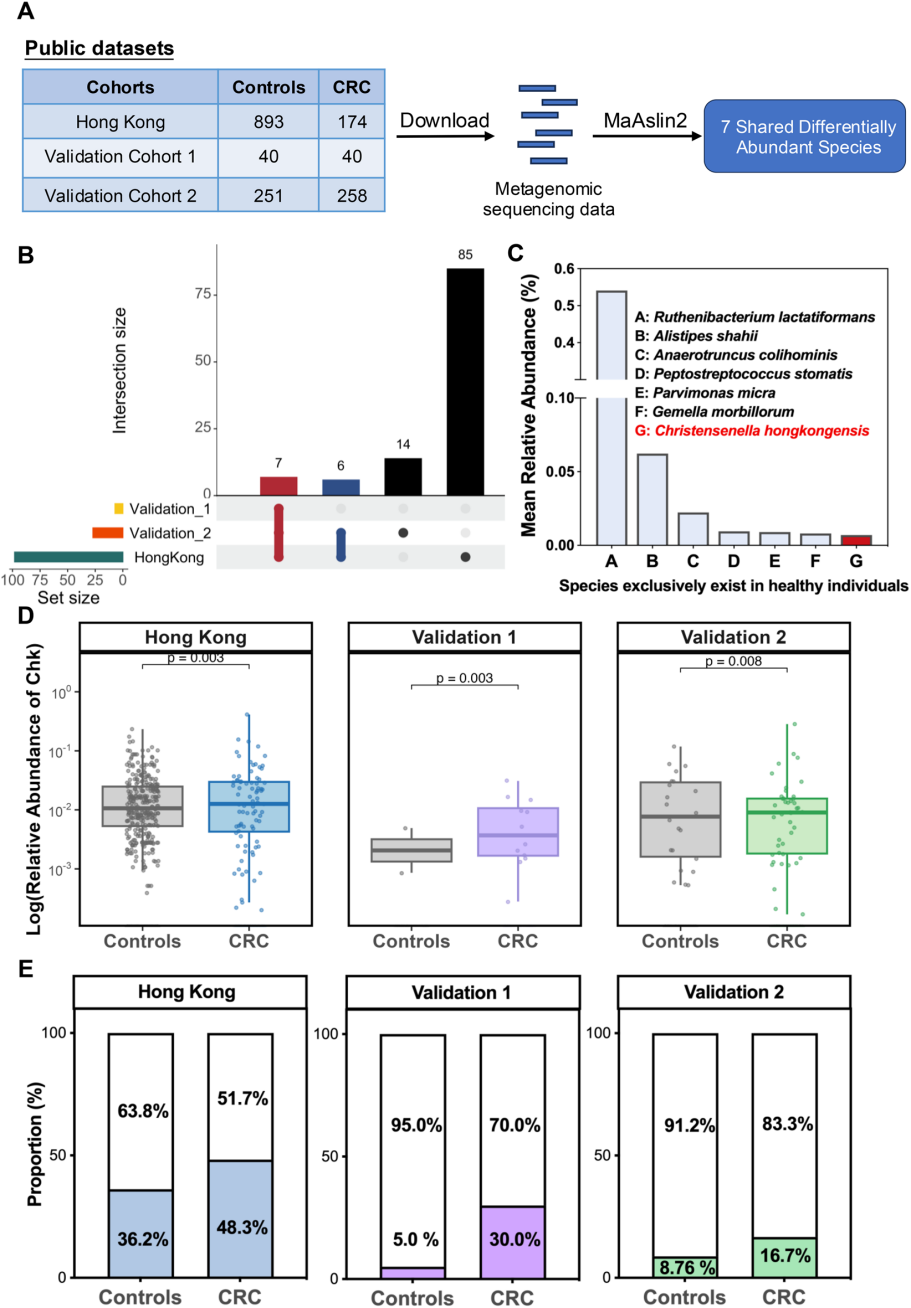
Identification of the novel species *C. hongkongensis* enriched in patients with CRC. **A** workflow of metagenomic analysis conducted using one in-house Hong Kong dataset and two public datasets. Differentially abundant species were identified across the three cohorts using MaAslin2. **B** upset plot showing 7 identified differential species shared across 3 cohorts identified by MaAslin2 analysis. **C** comparison of the overall relative abundance of these 7 species in healthy controls across the combined data from three cohorts. **D** comparison of the relative abundance of *C. hongkongensis* between CRC patients and healthy controls in each individual cohort. **E** comparison of the prevalence of *C. hongkongensis* between CRC patients and healthy controls in each cohort. Data are presented as mean±SEM, with statistical differences between groups assessed using the two-sided Mann-Whitney test. Sample sizes (**n**) are indicated at the base of each bar

To identify potential novel bacterial markers for CRC, we performed MaAsLin2 analysis on metagenomic sequencing data from our internal cohort and two public cohorts to determine differentially abundant bacterial species in fecal samples of CRC patients compared to healthy controls (Fig. 1A). Among all identified differential bacterial species, seven CRC-enriched species were consistently observed across all three cohorts, including *Ruthenibacterium lactatiformans*, *Alistipes shahii*, *Anaerotruncus colihominis*, *Peptostreptococcus stomatis*, *Parvimonas micra*, *Gemella morbillorum* and *Christensenella hongkongensis* (all *p* < 0.05, Fig. 1B and Fig. S1). Notably, *C. hongkongensis* demonstrated the lowest abundance in healthy controls among these seven species (Fig. 1C). In each individual cohort, the relative abundance of *C. hongkongensis* was also significantly increased in CRC patients compared to healthy controls (all *p* < 0.01, Fig. 1D). Furthermore, the prevalence of *C. hongkongensis* was consistently elevated in CRC patients compared to healthy controls across all three cohorts (Fig. 1E). Collectively, we identified a novel bacterial species that is consistently enriched in CRC across the cohorts.

### The involvement of *C. hongkongensis* in CRC progression from adenoma to carcinoma

**Fig. 2:**
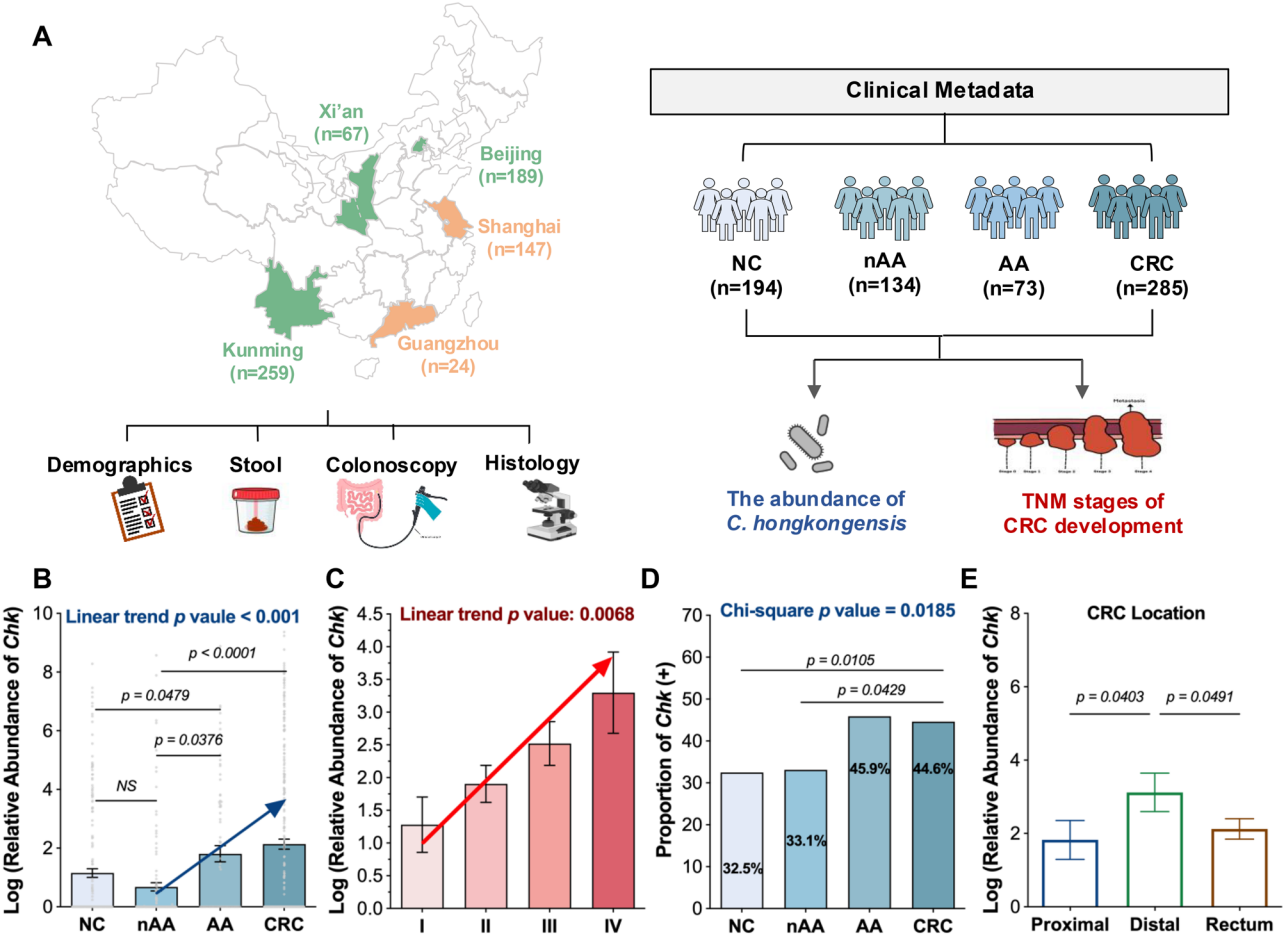
The involvement of *C. hongkongensis* in CRC progression from adenoma to carcinoma. **A** workflow illustrating the quantitation of *C. hongkongensis* abundance in patients with non-advanced adenoma (nAA), advanced adenoma (AA), CRC and healthy controls (NC) from mainland China. Clinical metadata, including demographics, fecal samples, colonoscopy results, and histology, were collected for analysis. **B** quantitative analysis of the relative abundance of *C. hongkongensis* among different groups (NC, nAA, AA, and CRC) from mainland China using qPCR. **C** linear regression analysis showing a positive association between the relative abundance of *C. hongkongensis* and TNM stages in CRC patients. **D** comparison of the prevalence of *C. hongkongensis* among nAA, AA, CRC and NC groups. **E** distribution of fecal *C. hongkongensis* levels across different colon locations (proximal colon, distal colon and rectum) in CRC patients. Data are presented as mean±SEM, *p* values were obtained from linear mixed-effects models with study center as a random effect. The chi-square test was used to evaluate the prevalence of *C. hongkongensis* across groups, and simple regression analyses were used to estimate the association between *C. hongkongensis* levels and factors of interest. NS, *p* > 0.05

To investigate the potential role of *C. hongkongensis* in CRC progression, we further quantitatively examined its abundance in an expanded cohort of fecal samples collected from healthy controls (NC, *n* = 194), patients with non-advanced adenomas (nAA, *n* = 134), patients with advanced adenomas (AA, *n* = 73) and those with CRC (*n* = 285) across five regions from mainland China (Guangzhou, Kunming, Beijing, Shanghai and Xi’an, *n* = 686) using our previously established duplex-qPCR platform (Fig. 2A) [5].

Consistent with previous findings, a substantially enriched abundance of *C. hongkongensis* was observed in CRC patients compared to healthy controls (*p* < 0.001, Fig. 2B). More importantly, the abundance of *C. hongkongensis* progressively increased along with the CRC progression, from the nAA stage to the AA stage, and ultimately to carcinoma (*p* < 0.001), with no significant differences detected between patients with nAA and healthy controls (Fig. 2B). Furthermore, among the CRC patients, the abundance of *C. hongkongensis* also demonstrated a continuous upward trend as the disease advanced from TNM stage I to TNM stage IV, reflecting a compelling correlation with tumor progression (*p* < 0.01, Fig. 2C). In addition to the abundance, the prevalence of *C. hongkongensis* also demonstrated a notable increase in CRC patients at the AA stage and those presenting with adenoma, in comparison to healthy controls and CRC patients at the nAA stage (NC: 32.5%; nAA: 33.1%; AA: 45.9%; CRC: 44.6%, Fig. 2D). However, no statistically significant differences were observed between CRC patients at the nAA stage and healthy controls (Fig. 2D). Notably, the abundance of *C. hongkongensis* exhibited a markedly elevated level in patients with distal CRC, compared to those with tumors located in proximal colon or rectal regions (both *p* < 0.05, Fig. 2E). These compelling findings suggested the potential of *C. hongkongensis* as an early fecal biomarker, providing a valuable approach for the early detection of precursory lesions that may lead to CRC, particularly in patients with distal CRC.

### Combination of *C. hongkongensis* and FIT improves the detection of advanced adenoma

**Fig. 3:**
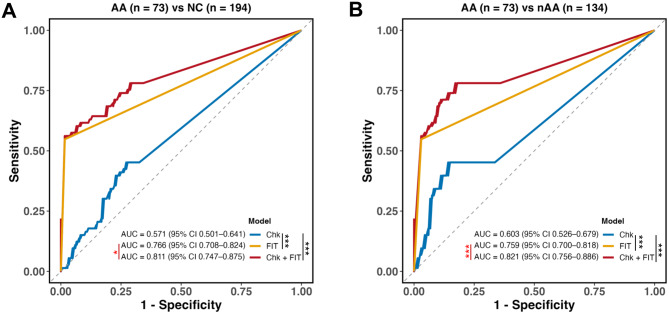
Diagnostic performance of *C. hongkongensis* and FIT in single and combined testing in the mainland china cohort. Comparison the diagnostic performance **A** between AA and NC groups; **B** between AA and nAA groups. For each comparison, ROC curves represent the diagnostic accuracy of fecal *C. hongkongensis* (Chk; blue lines), FIT (orange lines), and their combined model (Chk + FIT; red lines). The area under the curve (AUC) values, 95% confidence intervals (CIs), and significance levels are shown within each panel. ***p < 0.001; *p < 0.05; NS, *p* > 0.05

We next evaluated the diagnostic performance of *C. hongkongensis* using receiver operating characteristic (ROC) analysis in the mainland China cohort (Table 1 and Fig. 3). For differentiating AA from NC, the AUC was 0.57 (95%CI 0.50–0.64), with a sensitivity of 45.2% and specificity of 72.8% (Fig. 3A). Similar performance was observed for distinguishing AA from nAA, with an AUC of 0.60 (95% CI 0.53–0.68), sensitivity of 45.2% and specificity of 85.8% (Fig. 3B). *C. hongkongensis* also showed a modest discriminatory ability for CRC, with AUCs of 0.58 (95% CI 0.54–0.63, Fig. S3A) for CRC vs controls, and AUCs of 0.60 (95% CI 0.56–0.65. Fig. S3B) for CRC vs nAA, respectively.

**Table 1:** Diagnostic performance of FIT, *C. hongkongensis* (Chk) test, and the combined model (Chk + FIT) for detection of CRC and AA, compared with healthy controls or nAA

	Threshold	Sensitivity	Specificity	AUC	ppv	npv	95% CI	P value#
**AA vs NC model**								
Chk	1.12	45.2%	72.8%	0.57	0.40	0.77	0.50–0.64	<0.001
FIT	0.50	54.8%	98.4%	0.77	0.93	0.85	0.71–0.82	-
Chk+FIT	0.38	56.2%	98.4%	0.81	0.93	0.85	0.75–0.88	<0.05
**AA vs nAA model**								
Chk	1.25	45.2%	85.8%	0.60	0.64	0.74	0.53–0.68	<0.001
FIT	0.50	54.8%	97.0%	0.76	0.91	0.80	0.70–0.82	-
Chk+FIT	0.20	78.1%	82.8%	0.82	0.71	0.87	0.76–0.89	<0.001
**CRC vs NC model**								
Chk	0.97	42.8%	72.8%	0.58	0.71	0.45	0.54–0.63	<0.001
FIT	0.50	82.8%	98.4%	0.91	0.98	0.79	0.88–0.93	-
Chk+FIT	0.37	84.6%	97.8%	0.92	0.98	0.80	0.89–0.94	NS
**CRC vs nAA model**								
Chk	0.97	42.8%	84.3%	0.60	0.85	0.41	0.56–0.65	<0.001
FIT	0.50	82.8%	97.0%	0.90	0.98	0.73	0.87–0.93	-
Chk+FIT	0.70	83.2%	97.0%	0.91	0.98	0.73	0.89–0.94	NS

**Abbreviations:** AA, advanced adenoma; CRC, colorectal cancer; Chk, *C. hongkongensis*; FIT, fecal immunochemical test; NC, normal control; nAA, non-advanced adenoma; AUC, area under the ROC curve; PPV, positive predictive value; NPV, negative predictive value

# *p* values were obtained from pairwise comparisons of AUCs using Delong’s test (Chk vs FIT and Chk + FIT vs FIT). NS, not significant

Given that FIT is the stool-based screening method recommended by major international guidelines, its diagnostic performance was further evaluated in the same cohort. At a threshold of 34 μg Hb/g, FIT exhibited moderate accuracy for detecting advanced adenoma. Specifically, the AUC values were 0.77 (95% CI 0.71–0.82) for AA vs NC (Fig. 3A) and 0.76 (95% CI 0.70–0.82) for AA vs nAA (Fig. 3B), with a sensitivity of 54.8% and a specificity of 97.0%. For CRC detection, FIT demonstrated great diagnostic ability, with the AUCs of 0.91 (95% CI 0.88–0.92; CRC vs NC) and 0.90 (95% CI 0.87–0.93; CRC vs nAA), achieving high sensitivity (82.8%) and specificity (97.0–98.4%; Fig. S3). These results indicate that FIT serves as a highly specific tool for detecting bleeding-related lesions such as CRC, whereas its sensitivity for premalignant adenomas remains moderate.

To determine whether *C. hongkongensis* could enhance diagnostic performance in advanced adenoma detection, we constructed a combined model incorporating both *C. hongkongensis* and FIT. This integrated model substantially improved diagnostic accuracy for advanced adenoma, with AUCs significantly increasing from 0.77 to 0.81 for AA vs NC (*p* < 0.05) and from 0.76 to 0.82 for AA vs nAA (*p* < 0.001). The corresponding sensitivity increased from 54.8% to 56.2% and 78.1%, respectively, while specificity remained high (82.8%-98.4%, Fig. 3A–B).

In contrast, for CRC detection, the combination provided only marginal improvements (AUC changes < 0.02, *p* > 0.05, Fig. S3), indicating that FIT alone already achieves near-optimal diagnostic efficiency for overtly bleeding malignancies.

#### *C. hongkongensis* promotes CRC cell growth

**Fig. 4:**
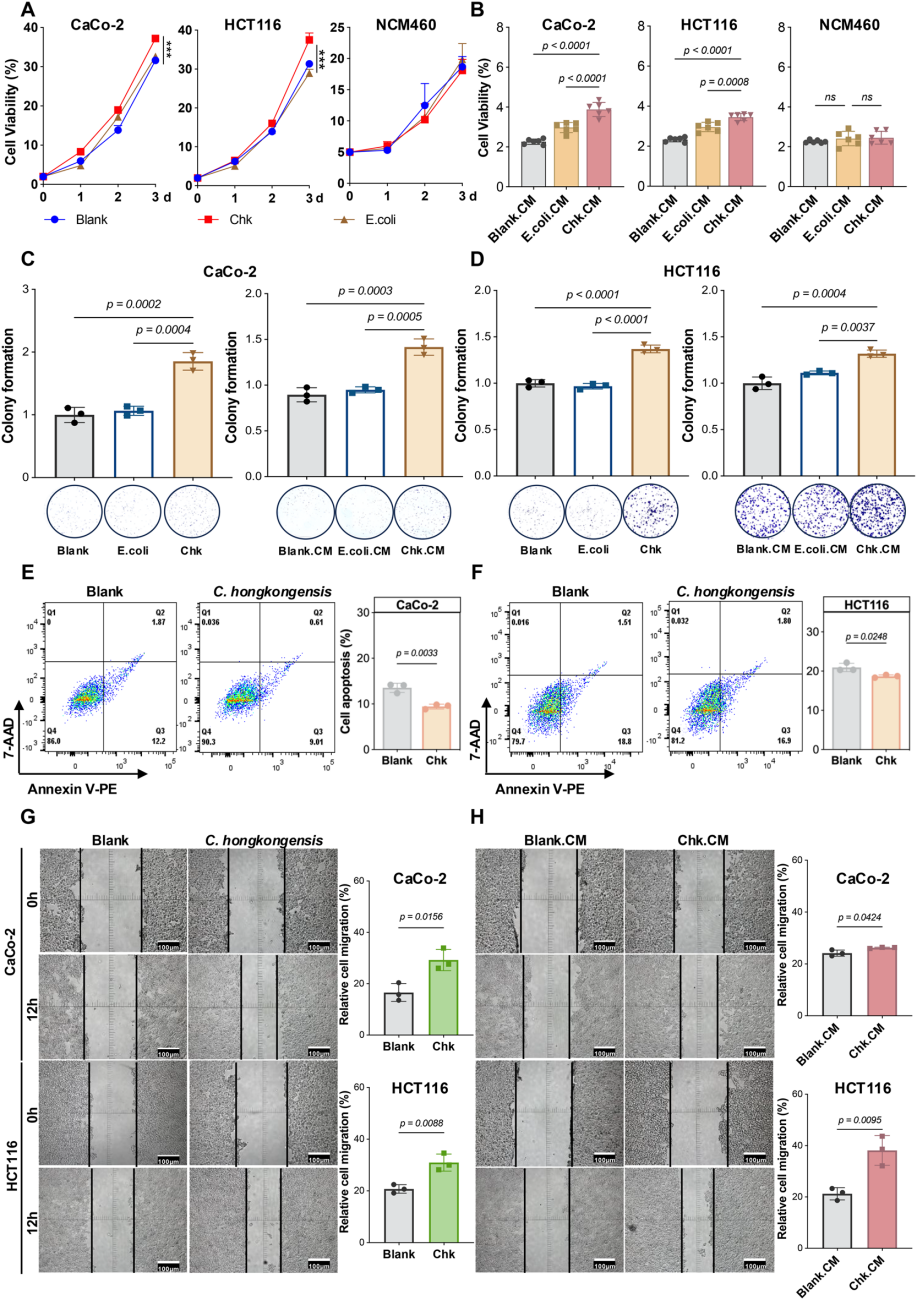
*C. hongkongensis* promotes CRC cell growth. **A** effects of live *C. hongkongensis* (MOI = 100) on the proliferation of CRC cells (CaCo-2, HCT116) and normal colon epithelial cells (NCM460). *E. coli *MG1655 was used as a negative control. **B** effects of *C. hongkongensis* conditioned medium (Chk.CM, 5% *v/v*) on CRC cell viability, compared to *E. coli* conditioned medium (E. coli.CM) and peptone yeast extract glucose (PYG) medium as blank controls. **C** and **D** colony formation assays showing the effects of live *C. hongkongensis* and Chk.CM on CaCo-2 (C) and HCT116 (D) cells compared to blank controls. **E** and **F** cell apoptosis analysis in CaCo-2 (E) and HCT116 (F) cells using Annexin V/7-AAD staining and flow cytometry after 48 hours of co-culture with live *C. hongkongensis* (MOI = 100, 4-hour infection). **G** and **H** wound healing assay results showing the migration rates of CaCo-2 and HCT116 cells after 12 hours of treatment with either live *C. hongkongensis* (G) or 5% Chk.CM (H). Data are presented as mean±SD. Statistical significance was determined by two-way ANOVA (A), one-way ANOVA with Fisher’s LSD test (B, C and D), and unpaired t-test (E, G and H). *p < 0.05, **p < 0.01, ***p < 0.001; NS, *p* > 0.05

The progressively increased abundance of *C. hongkongensis* during CRC progression suggests that *C. hongkongensis* may play a carcinogenic role in CRC tumorigenesis. To prove this, we conducted the in vitro experiments using two CRC cell lines (CaCo-2 and HCT116) and a normal colon epithelial cell line (NCM460), co-cultured with *C. hongkongensis* and its conditioned medium. *E. coli* MG1655, a non-pathogenic strain of *E. coli*, was employed as a negative control. Both live *C. hongkongensis* and its 5% conditioned medium demonstrated the ability in enhancing the proliferation of two CRC cell lines (live *C. hongkongensis*: both *p* < 0.001; 5% conditioned medium: both *p* < 0.0001), yet neither exerted any influence on the growth of NCM460 cells (Fig. 4A and 4B). In keeping with this, the number of colonies which had formed in CaCo-2 and HCT116 cells cultured with *C. hongkongensis* or its conditioned medium were increased significantly compared with those treated with PBS or *E. coli* MG1655 (Fig. 4C and 4D).

In addition, flow cytometry utilizing dual staining with Annexin V-PE and 7-AAD was conducted to evaluate the effect of *C. hongkongensis* on cell apoptosis. The result exhibited an increase in the number of apoptotic cells in *C. hongkongensis* treated CaCo-2 cells compared with those treated with PBS (9.43±0.52% vs 13.53 ± 1.01%, *p* < 0.01, Fig. 4E). A similar effect was observed in *C. hongkongensis* cultured HCT116 cells, which demonstrated an increased proportion of cells in the apoptotic phase compared with cells co-cultured with PBS (18.69±0.36% vs 20.93 ± 1.05%, *p* < 0.05, Fig. 4F).

Furthermore, the monolayer wound healing assay was performed to investigate the effects of *C. hongkongensis* on CRC cell migration. Administration of either *C. hongkongensis* or its conditioned medium significantly enhanced the cell migration capacity in both CaCo-2 and HCT116 cell lines (Fig. 4G and 4H). Quantitative analyses at 12 hours post-wounding confirmed a significant increase in wound closure in cells administered with *C. hongkongensis* or its conditioned medium compared to control cells (29.22% and 26.25% in CaCo-2 cells; 30.95% and 38.15% in HCT116 cells, respectively), thereby suggesting that the migration rate of cells cultured with *C. hongkongensis* or its conditioned medium was significantly higher than that of the control cells. Collectively, these data suggest that *C. hongkongensis* and its conditioned medium exert pro-tumorigenic effects on CRC cells by enhancing proliferation, colony formation, and migration, while simultaneously inhibiting apoptosis.

#### *C. hongkongensis* activates the tumorigenic signaling pathways in CRC

To further elucidate the molecular mechanisms mediated by *C. hongkongensis* in executing its oncogenic functions, we conducted transcriptomic sequencing to identify the genes and signaling pathways regulated by live *C. hongkongensis* and its conditioned medium in CaCo-2 and HCT116 cells. Principal component analysis (PCA) demonstrated a clear separation among all three treatment groups in HCT116 cells (Fig. S4A), whereas cells treated with live *C. hongkongensi*s and its conditioned medium clustered closely together in CaCo-2 cells, yet remained distinctly separated from the blank controls (Fig. S4B).

**Fig. 5:**
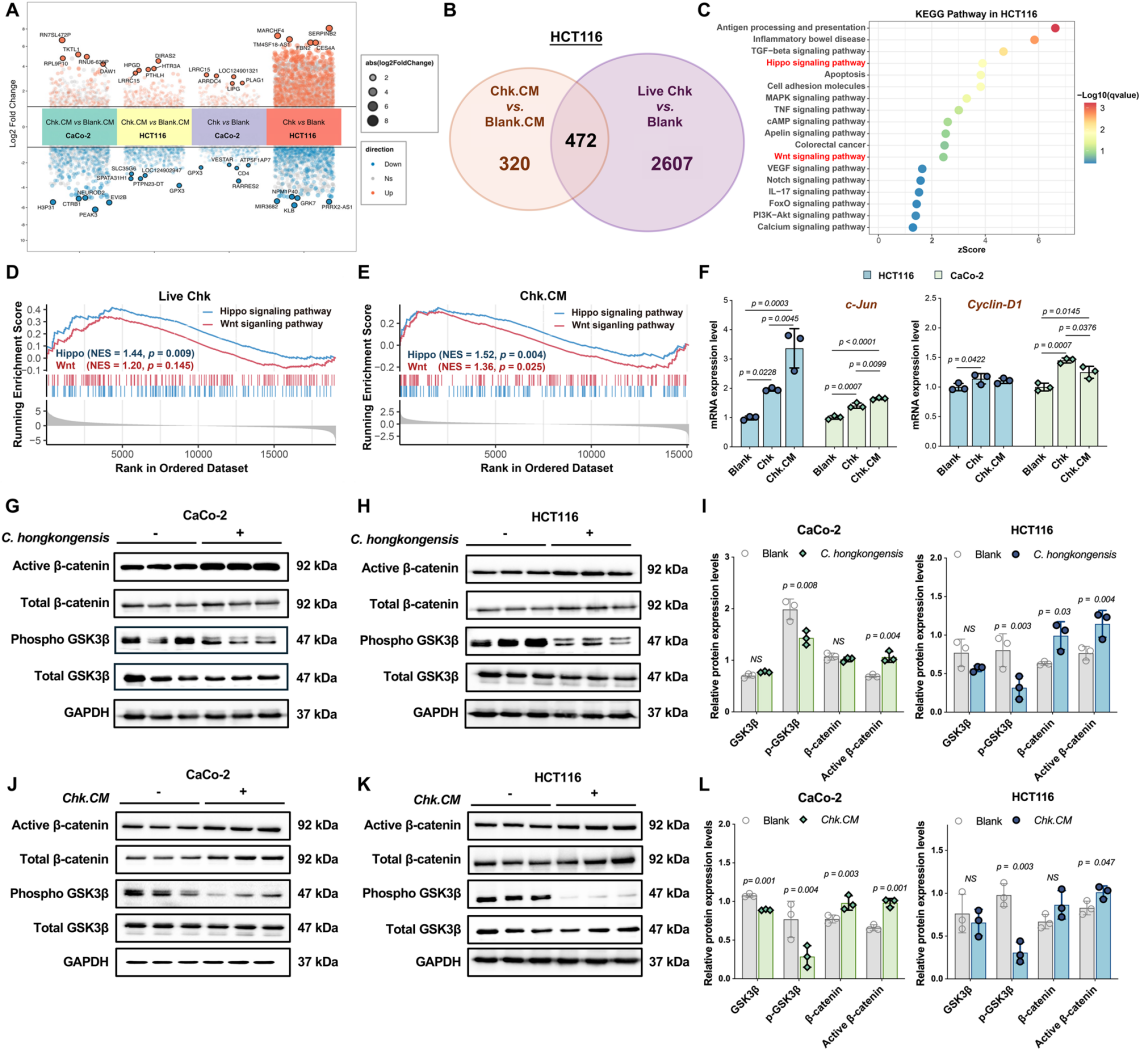
*C. hongkongensis* activates the tumorigenic signaling pathways in CRC. **A** multi-group volcano plot displaying significantly upregulated and downregulated genes in HCT116 and CaCo-2 cells after treatment with live *C. hongkongensis* or 5% Chk.CM. **B** venn diagram showing the intersecting differentially expressed genes (DEGs) between live *C. hongkongensis* and 5% Chk.CM treatments in HCT116 cells. **C** bubble plot illustrating KEGG pathway enrichment of these intersecting DEGs in HCT116 cells. **D** and **E** GSEA enrichment plots for HCT116 cells co-cultured with live *C. hongkongensis* (D) or 5% Chk.CM (E), showing significant enrichment of the Hippo and Wnt signaling pathway. Normalized enrichment scores (NES) and *p*-values are indicated. **F** the mRNA expression level of Wnt/β-catenin signaling pathway (*c-Jun, and Cyclin-D1*) in HCT116 and CaCo-2 cells after different treatments. (*n* = 3). **G** and **H** *C. hongkongensis* activated Wnt/β-catenin signaling pathway in CaCo-2 (G) and HCT116 cells (H) after co-culture for 4 h (*n* = 3). **I** the protein expression level of GSK3β, phosphorylated GSK3β, β-catenin and active β-catenin in CaCo-2 and HCT116 cells after co-culture with *C. hongkongensis*. **J** and **K** 5% Chk.CM activated Wnt/β-catenin signaling pathway in CaCo-2 (J) and HCT116 (K) cells (*n* = 3). **L** the protein expression level of GSK3β, phosphorylated GSK3β, β-catenin and active β-catenin in CaCo-2 and HCT116 cells after co-culturing with 5% Chk.CM. Data are presented as mean±SD. Unpaired twosided *t*-test was used for comparisons in I and L. One-way ANOVA with the Turkey’s post hoc test was performed for F. NS, *p* > 0.05

The differentially expressed genes (DEGs) between *C. hongkongensis*-treated and PBS-treated CRC cells were identified using DESeq2 (Fig. 5A). In CaCo-2 cells, co-culturing with live *C. hongkongensis* resulted in the upregulation of 203 genes and downregulation of 223 genes. While treatment with its conditioned medium significantly altered the expression of 939 genes, including 313 upregulated and 626 downregulated (Fig. 5A). Similarly, in HCT116 cells, treatment with live *C. hongkongensis* led to the upregulation of 1960 genes and downregulation of 1119 genes, whereas treatment with its conditioned medium resulted in 324 upregulated and 468 downregulated genes (Fig. 5A). Among all DEGs, 290 genes in CaCo-2 cells and 472 genes in HCT116 cells were consistently regulated in both live *C. hongkongensis*-treated and conditioned medium-treated cells compared to the control groups (Fig. S4C and Fig. 5B). Following KEGG pathway enrichment analysis, the Wnt signaling pathway and the Hippo signaling pathway were identified as activated in both CRC cell lines treated with live *C. hongkongensis* or its conditioned medium (Fig, 5C and Fig. S4D). Moreover, gene set enrichment analysis (GSEA) further confirmed that the Hippo signaling pathway and the Wnt signaling pathway were significantly upregulated in HCT116 cells treated with either live *C. hongkongensis* (Hippo signaling pathway: normalized enrichment score [NES]=1.44, *p* < 0.01; Wnt signaling pathway: NES = 1.52, *p* > 0.05) or its conditioned medium (Hippo signaling pathway: NES = 1.20, *p* < 0.01; Wnt signaling pathway: NES = 1.36, *p* < 0.05, Fig. 5D and 5E). Additionally, PathView analysis visualized the gene expression changes in both Hippo signaling pathway and Wnt signaling pathway, revealing that activation of the Wnt/β-catenin signaling pathway represents a shared mechanism underlying the response in both cell lines (Fig. S5 and S6).

The Wnt/β-catenin signaling pathway serves as a critical regulator of intestinal homeostasis, governing essential cellular processes such as proliferation, differentiation, and migration, and has been strongly associated with the initiation and progression of CRC. Activation of the Wnt/β-catenin pathway typically results in accumulation of active β-catenin and concomitant inhibition of GSK3β phosphorylation, leading to transcriptional activation of downstream oncogenic targets. Western blot results showed that either co-culture with live *C. hongkongensis* (Fig. 5I) or its conditioned medium (Fig. 5L) significantly increased the protein level of active β‑catenin (*p* < 0.05), while reducing that of phosphorylated GSK3β (*p* < 0.01) in both CaCo‑2 (Fig. 5G and 5J) and HCT116 cells (Fig. 5H and 5K). At the transcriptional level, the mRNA expression of canonical downstream Wnt/β-catenin target genes (*CyclinD1* and *cJun)* was significantly increased in both cell lines after co-culture with *C. hongkongensis* or its conditioned medium (Fig. 5F). These results therefore indicate that *C. hongkongensis* exerts its oncogenic function in CRC cells through the activation of Wnt/β-catenin signaling pathway.

### The conditioned medium of *C. hongkongensis* enhances growth of CRC patient-derived organoids *via *activation of Wnt/β-catenin signaling pathway

**Fig. 6:**
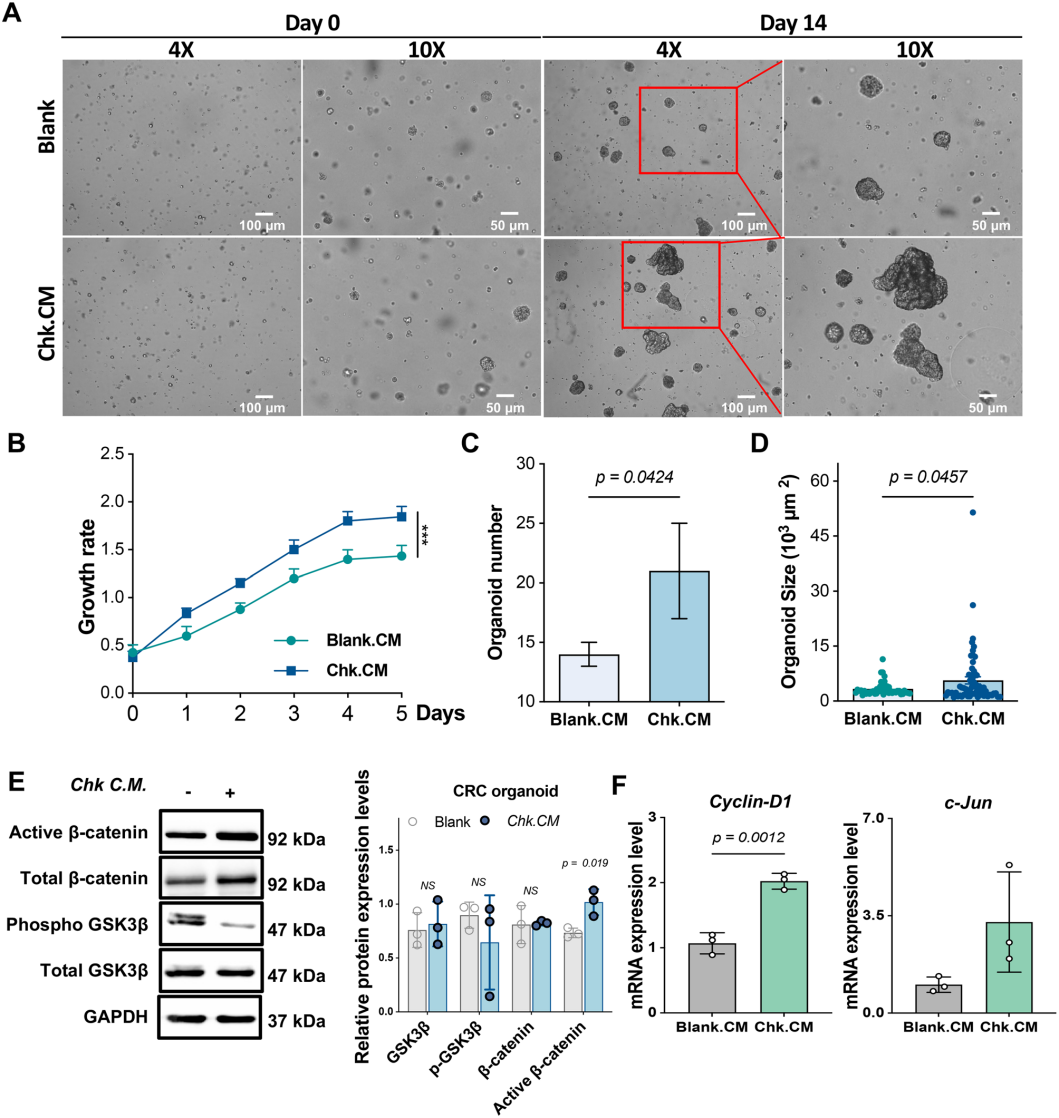
The conditioned medium of *C. hongkongensis* enhances growth of CRC patient-derived organoids *via* activation of Wnt/β-catenin signaling pathway. **A** representative images of CRC organoids co-cultured with *C. hongkongensis* conditioned medium (Chk.CM, 5% *v/v*) at Day 0 (D0) and Day 14 (D14). **B** proliferation curve showing the growth rate of CRC organoids treated with Chk.CM over 5 days. **C** quantification of organoid number after treatment with Chk.CM at Day 14. **D** organoid size was quantified at Day 14. **E** Chk.CM activated Wnt/β-catenin signaling pathway in CRC organoids. The right panel shows protein expression levels of GSK3β, phosphorylated GSK3β, β-catenin and active β-catenin (*n* = 3). **F** the mRNA expression levels of genes involved in Wnt/β-catenin signaling pathway (*c-Jun*, and *Cyclin-D1*) in CRC organoids after co-culture with Chk.CM (*n* = 3). Data are presented as mean±SD. Statistical significance was determined by two-way ANOVA (B) and unpaired t-test was performed (A, C, D, E). ***p < 0.001; NS *p* > 0.05

The activation of the Wnt/β-catenin signaling pathway triggered by *C. hongkongensis* was further validated in CRC organoid derived from one CRC patient. The organoids were co-cultured with *C. hongkongensis* conditioned medium or fresh PYG medium. As shown in Fig. 6A and 6D, the CRC organoids treated with the conditioned medium of *C. hongkongensis* exhibited a significantly greater size compared to the blank control (*p* < 0.05). Moreover, administration of the conditioned medium of *C. hongkongensis* significantly enhanced the proliferation rate of CRC organoids during the 5-day cultivation period (*p* < 0.001, Fig, 6B), and the number of organoids was higher than that in the blank control group (*p* < 0.05, Fig. 6C). At the protein level, 5% conditioned medium of *C. hongkongensis* led to activation of the Wnt/β-catenin signaling pathway (Fig. 6E), as evidenced by elevated protein levels of active β-catenin (*p* < 0.05) and reduced phosphorylation of GSK3β. Consistent with these results, the mRNA expression levels of *Cyclin-D1* (*p* < 0.01) and *c-Jun* were increased in CRC organoids treated with the conditioned medium of *C. hongkongensis* compared to the blank control group (Fig. 6F). These findings suggest that the secretome of *C. hongkongensis* is capable of enhancing the growth of CRC patient-derived organoids by activating the Wnt/β-catenin signaling pathway.

## Discussion

Due to the high cost and invasive nature of standard diagnostic methods like colonoscopy and sigmoidoscopy, there is an urgent need to improve the sensitivity of non-invasive tests for detecting CRC [35]. Increasing evidence has revealed that alterations in gut microbiota composition are closely associated with CRC progression, suggesting that the gut microbiome represents a rich reservoir of potential biomarkers [8]. Among various approaches, shotgun metagenomic sequencing, due to its high taxonomic precision and functional resolution, has been widely employed to identify CRC associated microbial markers [36].

In this study, we analyzed fecal metagenomic sequencing data from our internal cohort and two public cohorts by MaAsLin2, adjusting for major covariates including age, sex, and BMI. Seven species were significantly enriched in CRC patients compared to healthy controls. Among these, six species including *Ruthenibacterium lactatiformans* [37], *Alistipes shahii* [38], *Anaerotruncus colihominis* [39], *Peptostreptococcus stomatis* [40], *Parvimonas micra* [35] and *Gemella morbillorum* [41], have been reported as potential CRC biomarkers with varying diagnostic performances [42]. Notably, *C. hongkongensis* was distinguished by its lowest abundance in healthy controls and significant enrichment in CRC patients. Consistent with our findings, multi-omics analysis of CRC patients revealed that *Christensenella* abundance continuously increased from early-stage colonic intramucosal carcinoma to advanced CRC stages [43], further supporting the strong association of *C. hongkongensis* with CRC development and its potential as a microbial marker.

Early detection during precancerous stages is critical for reducing CRC incidence and mortality [44]. However, most known biomarkers, including microbial and genetic markers, primarily differentiate colorectal adenomas or CRC from healthy controls, showing limited ability to distinguish among precancerous stages. For example, *Fusobacterium nucleatum,* a well-characterized pathogen associated with carcinogenic activity in CRC, demonstrated the diagnostic performance with an AUC of 0.59 in differentiating advanced adenoma from controls [45], comparable to the accuracy of *C. hongkongensis*, which achieved an AUC of 0.57. Additionally, *F. nucleatum* showed no significant differences between patients with advanced adenoma and controls [45]. To our knowledge, this is the first study to report the performance of single-species biomarkers in differentiating early-stage adenoma (non-advanced adenoma) from advanced adenoma or CRC. In the study by *Yachida et al*., a multivariate model incorporating 29 bacterial species achieved an AUC of 0.73 for differentiation between earlystage CRC (TNM stage 0) and controls [46]. However, the AUC for individual species was not reported. Similarly, while the methylated gene SHOX2 has been proposed as a biomarker for CRC diagnosis, quantitative methylation analysis reveals no significant differences in SHOX2 methylation levels between nAA and AA patients [47]. These findings highlight that *C. hongkongensis* as a promising early microbial marker for detecting high-risk lesions.

Recent studies have demonstrated that combining bacterial biomarkers with FIT can significantly enhance adenoma detection [35, 48]. For example, *Wong et al.* reported that supplementing FIT with *F. nucleatum* improved diagnostic performance for advanced adenoma detection (AUC = 0.65) [5]. Given this evidence, we further evaluated the diagnostic performance of *C. hongkongensis* in combination with FIT. This combination significantly improved advanced adenoma detection, raising AUCs to 0.82 for AA vs NC (Fig. 3A) and 0.81 for AA vs nAA (Fig. 3B). These findings suggest that incorporating *C. hongkongensis* quantification into FIT-based screening strategies could enhance the early identification of high-risk individuals.

While our study focused on participants from mainland China, *C. hongkongensis* shows promise for broader application. Beyond our cohort, *C. hongkongensis* has been detected in human gut microbiota and clinical cases across Asia, Europe, North America, and Oceania [49]. Consistently, a recent global multicenter metagenomic analysis reported that *C. hongkongensis* exhibited higher abundance in left-sided CRC [50], suggesting that its association with colorectal neoplasia may extend across different ethnic and geographic backgrounds. However, detailed metadata on diet, medication use, and ethnicity were not available in this study, therefore residual confounding could not be fully assessed. The widespread detection of *C. hongkongensis* in populations with different lifestyles and medication patterns may suggest that these hostrelated factors are unlikely to be the primary determinants of its enrichment in CRC. Future larger multicenter and multiethnic validation studies are needed to confirm its global applicability as a microbial biomarker for CRC screening.

Previous studies have reported both pathogenic and probiotic roles within the *Christensenellaceae* family. On the one hand, *Christensenellaceae* was significantly enriched in CRC patients compared with healthy controls [51], implying its involvement in CRC-related microbial dysbiosis. Specifically, using linear discriminant analysis (LDA), *Christensenellaceae* was identified as positively associated with CRC in African-American patients [52], reflecting possible ethnicity-dependent microbial patterns. Consistently, another study reported that alterations in *Christensenellaceae* abundance were associated with CRC-related host gene mutations, specifically APC and ZNF717 [53], suggesting a potential host–microbe genomic interaction relevant to tumorigenesis. Collectively, these studies indicate that shifts in *Christensenellaceae* abundance are frequently linked to CRC occurrence and are influenced by host genetic and demographic factors. On the other hand, in contrast to these observations, other studies have revealed that higher *Christensenellaceae* in human intestinal tract is positive associated with host health [16]. These discrepancies suggest that different species within the *Christensenellaceae* family may exert distinct, possibly opposing, effects on CRC, highlighting the need for further species-level identification and functional characterization.

Our in vitro studies revealed that *C. hongkongensis* and its conditioned medium enhance CRC cell proliferation and migration, while simultaneously inhibiting apoptosis (Fig. 4). To better understand these effects, we compared the characteristics of *C. hongkongensis* with other reported species of the *Christensenella* genus. Fundamentally, although belonging to the same genus, *C. hongkongensis* is a Gram-positive bacterium, whereas *Christensenella minuta* and *Christensenella intestinihominis* are Gram-negative bacteria, highlighting a fundamental phenotypic distinction between *C. hongkongensis* and other *Christensenella* species. Additionally, unlike *C. minuta* and *C. intestinihominis*, commensal bacteria commonly enriched in the healthy gut and known to contribute to antiinflammatory activity and metabolic homeostasis [18, 54], *C. hongkongensis* is frequently isolated from bacteremia cases, supporting its classification as an opportunistic pathogen [55]. From a functional perspective, *C. intestinihominis* is capable of producing short-chain fatty acids that induce GLP-1 secretion [56], whereas comparative genomic and metabolic analyses indicate that *C. hongkongensis* produces fewer beneficial metabolites [57]. Therefore, these differences in phenotypic characteristics, ecological niches, and metabolic capacities may explain the functional discrepancies between *C. hongkongensis* and other *Christensenella* species, underlie the contrasting effects on the host, and partly account for the tumor-promoting potential of *C. hongkongensis* in CRC.

Mechanistically, a network analysis based on pathway interaction database (PID) revealed a positive correlation between *Christensenellaceae* and the Wnt/β-catenin signaling pathway in mutant CRC patients [53]. Consistently, we found that *C. hongkongensis* and its conditioned medium promote CRC progression by activating the Wnt/β-catenin signaling pathway (Fig. 5). This activation leads to the nuclear accumulation of β-catenin and subsequent transcription upregulation of oncogenic genes such as *Cyclin-D1* and *c-Jun*, ultimately resulting in uncontrolled cell proliferation and tumor growth. However, the specific responsible metabolite(s) produced from *C. hongkoingensis* remain unidentified. Wnt signaling can be activated by bacteria through multiple mechanisms, including hijacking the pathway *via* direct injection of bacterial secreted proteins into host cells, modulation of the pathway by bacterial metabolites, and activation of the pathway through infection-induced signaling cascades [58–60]. Additionally, given the complexity of bacterial secretomes, which include proteins, peptides, enzymes, and toxins in addition to metabolites, it is highly likely that Wnt signaling activation is not mediated solely by metabolites. Furthermore, since Wnt/β-catenin signaling can integrate multiple upstream signals, we hypothesize that the observed activation is more likely driven by the combined and potentially synergistic effects of multiple compounds secreted by *C. hongkongensis* rather than by a single effector.

Both in vitro and ex vivo experiments have demonstrated that *C. hongkongensis* and its conditioned medium promote CRC progression through activation of the Wnt/β-catenin signaling pathway. Furthermore, the fecal abundance of *C. hongkongensis* gradually increases during CRC progression, from the groups of normal individuals to the subjects with adenoma and eventually those with carcinoma. Notably, an elevated abundance is already observed at the non-advanced adenoma stage; therefore, our current findings highlight a potential pro-tumorigenic role for *C. hongkongensis* in CRC. However, whether it serves as a causative agent in colorectal cancer initiation remains elusive. To date, direct causal links between individual gut microbes and colorectal carcinogenesis have been established for only a few well-characterized pathobionts, such as *Fusobacterium nucleatum* [8, 61]. Future studies using germ-free or gnotobiotic mice will be useful to dissect the interactions between host and gut microbiota to determine whether *C. hongkongensis* alone or in microbial consortia accelerates CRC progression in vivo.

## Conclusions

In summary, this study characterizes a novel gut bacterium *C. hongkongensis* that increases in abundance from the adenoma-to-carcinoma sequence, highlighting its potential as an auxiliary marker for CRC, with potential applications in early detection, disease staging, and tumor localization. Mechanistically, *C. hongkongensis* promotes CRC progression *via* the Wnt/β-catenin signaling pathway, providing new insights into its pathogenic role. Future studies integrating animal models, metabolite profiling, and therapeutic interventions are warranted to fully elucidate the clinical and biological significance of *C. hongkongensis* in CRC.

## Electronic supplementary material

Below is the link to the electronic supplementary material.

Supplementary material 1

Supplementary material 2

Supplementary material 3

Supplementary material 4

Supplementary material 5

## Data Availability

The raw data of the RNA-seq analysis were deposited in the SRA database (PRJNA1280769). The data in this study are available within the article and related supplementary information files, or by inquiring the corresponding authors.

## Abbreviations

CRC: Colorectal cancer
TNM: Tumor node metastasis
FIT: Fecal immunochemical test
DEGs: Differentially expressed genes
GSEA: Gene set enrichment analysis
NES: Normalized enrichment score
nAA: Non-advanced adenoma
AA: Advanced adenoma
T2D: Type 2 diabetes
EMT: Epithelial-mesenchymal transition
PYG: Peptone-Yeast Extract-Glucose
FBS: Fetal bovine serum
CCK8: Cell counting kit 8
