# Supplementary material for: Discovery and characterization of Christensenella hongkongensis as a novel bacterium in the adenoma-carcinoma progression
Source: J Transl Med. 2026 Feb 28;24:468. doi: 10.1186/s12967-026-07886-9 (PMC13049741; doi:10.1186/s12967-026-07886-9)
Supplement: Supplementary file 1 — Supplementary material 1 [file 12967_2026_7886_MOESM1_ESM.docx]

Supplementary Table 1: The characteristics of all public datasets.

|  |  | Discovery Cohort | | Validation Cohort 1 | | Validation cohort 2 | |
| --- | --- | --- | --- | --- | --- | --- | --- |
|  |  | CRC | CTR | CRC | CTR | CRC | CTR |
| N |  | 174 | 893 | 40 | 40 | 258 | 251 |
| Gender | Female | - | - | 19 | 16 | 97 | 115 |
|  | Male | - | - | 21 | 24 | 161 | 136 |
|  | p value^$^ | - | | 0.49 | | 0.07 | |
| Age | Mean | - | - | 59.05 | 63.23 | 62.7 | 60.8 |
|  | SD | - | - | 12.83 | 12.17 | 9.6 | 12.6 |
|  | p value^#^ | - | | 0.14 | | 0.33 | |
| BMI | Mean | - | - | 23.08 | 22.36 | 23 | 22.7 |
|  | SD | - | - | 2.40 | 2.72 | 3.3 | 3 |
|  | p value | - | - | 0.21 | | 0.20 | |
| Subject Name |  |  |  | ThomasAM_2019_c | | YachidaS_2019 | |
| Country |  | Hong Kong | | Japan | | Japan | |

$ two-sided fisher exact test

#: two-sided Wilcox test
