# Supplementary material for: Discovery and characterization of Christensenella hongkongensis as a novel bacterium in the adenoma-carcinoma progression
Source: J Transl Med. 2026 Feb 28;24:468. doi: 10.1186/s12967-026-07886-9 (PMC13049741; doi:10.1186/s12967-026-07886-9)
Supplement: Supplementary file 3 — Supplementary material 3 [file 12967_2026_7886_MOESM3_ESM.docx]

Supplementary Table 4: Primer sequences used in this study.

| Organism | Amplicon | Primer (5’→3’) | Sequence |
| --- | --- | --- | --- |
| Bacteria | *C. hongkongensis* | Forward | GGGGCTTGAATCCGTTCTTA |
|  |  | Reverse | GCTTTGCAACCAAAAGTCCA |
|  | 16S | Forward | CGTCAGCTCGTGYCGTGAG |
|  |  | Reverse | CGTCRTCCCCRCCTTCC |
| Human | β-actin | Forward | CTCACCATGGATGATGATATCGC |
|  |  | Reverse | GGAATCCTTCTGACCCATGCC |
|  | Ctnb1 | Forward | TGTGAATCCCAAGTACCAGTGT |
|  |  | Reverse | CGTCAGACAAAGGAGAAACATT |
|  | Wnt11 | Forward | CGATGCTCCTATGAAGGTGAAA |
|  |  | Reverse | CTTCCGTTGGATGTCTTGTTG |
|  | Axin2 | Forward | TGTCTTAAAGGTCTTGAGGGTTGAC |
|  |  | Reverse | CAACAGATCATCCCATCCAACA |
|  | Tcf1 | Forward | GTGCTGCTGCAGGTAGGACT |
|  |  | Reverse | CCATCCTCAAAGAGCTGGAG |
|  | Cyclin D1 | Forward | GGATGCTGGAGGTCTGCGA |
|  |  | Reverse | TAGAGGCCACGAACATGCAAGT |
|  | c-jun | Forward | GTCCTTCTTCTCTTGCGTGG |
|  |  | Reverse | GGAGACAAGTGGCAGAGTCC |

Supplementary Table 4: Probe sequences used in this study.

| Organism | Amplicon | 5‘Fluorophore | Probe Sequence (5’→3’) | 3’ Fluorophore |
| --- | --- | --- | --- | --- |
| Bacteria | *C. hongkongensis* | 5’-FAM | CCTTGCAGCCGGCGCGTTCGT | 3’-TAMRA |
|  | 16S | 5’-VIC | TTAAGTCCCRYAACGAGCGCAACCC | 3’-TAMRA |
