# Supplementary material for: Discovery and characterization of Christensenella hongkongensis as a novel bacterium in the adenoma-carcinoma progression
Source: J Transl Med. 2026 Feb 28;24:468. doi: 10.1186/s12967-026-07886-9 (PMC13049741; doi:10.1186/s12967-026-07886-9)
Supplement: Supplementary file 4 — Supplementary material 4 [file 12967_2026_7886_MOESM4_ESM.docx]

Supplementary Table 5: Antibodies Used in this study.

| Antibody | Catalog | Company | Dilution | Source |
| --- | --- | --- | --- | --- |
| Phospho-GSK-3β (Ser9) (5B3) Monoclonal Antibody | 9323 | CST | 1:1000 | Rabbit |
| GSK-3β Monoclonal Antibody | 9315 | CST | 1:1000 | Rabbit |
| β-Catenin Antibody | 9562 | CST | 1:1000 | Rabbit |
| Active β-Catenin (Ser33/37/Thr41) Monoclonal Antibody | 8814 | CST | 1:1000 | Rabbit |
| GAPDH (14C10) Monoclonal Antibody | 2188 | CST | 1:1000 | Rabbit |
| Anti-Rabbit IgG, HRP-linked Antibody | G21234 | Thermo | 1:5000 | Rabbit |
