# Supplementary material for: Discovery and characterization of Christensenella hongkongensis as a novel bacterium in the adenoma-carcinoma progression
Source: J Transl Med. 2026 Feb 28;24:468. doi: 10.1186/s12967-026-07886-9 (PMC13049741; doi:10.1186/s12967-026-07886-9)
Supplement: Supplementary file 5 — Supplementary material 5 [file 12967_2026_7886_MOESM5_ESM.pdf]

Discovery and characterization of *Christensenella hongkongensis* as a novel bacterium in the adenoma-carcinoma progression

Wenqing Zhang [1,2], Qi Su [1,2], Haiyun Shi [3], Yang Sun [4], Xiaobo Li [5], Mengbin Li [6], Hui Wang [7], Jun Yu [2,8], Nathalie Wong [9], Francis K L Chan [1,8,10], Jingwan Zhang[#1,2], Siew C Ng [#1,2,8,11]

[1]Microbiota I-Center, Hong Kong SAR, China

[2]Department of Medicine and Therapeutics, The Chinese University of Hong Kong, Hong Kong SAR, China

[3]Beijing Friendship Hospital, Capital Medical University, Beijing, China

[4]The First Affiliated Hospital of Kunming Medical University, Kunming, Yunnan, China

[5]Renji Hospital, Shanghai Jiao Tong University School of Medicine, Shanghai, China

[6]Xijing Hospital, Xi'an, Shaanxi, China

[7]The Sixth Affiliated Hospital, Sun Yat-sen University, Guangzhou, Guangdong, China

[8]State Key Laboratory of Digestive Disease, Li Ka Shing Institute of Health Sciences, The Chinese University of Hong Kong, Hong Kong SAR, China

[9]Department of Surgery, Sir Y.K. Pao Centre for cancer, The Chinese University of Hong Kong, Shatin, Hong Kong SAR, China.

[10]Centre for Gut Microbiota Research, The Chinese University of Hong Kong, Hong Kong SAR, China

[11]New Cornerstone Science Laboratory, The Chinese University of Hong Kong, Hong Kong SAR, China

[#]**Correspondence:**

Siew Chien Ng, PhD, Department of Medicine and Therapeutics, The Chinese University of Hong Kong, 9/F, Lui Che Woo Clinical Sciences Building, Prince of Wales Hospital, Shatin, Hong Kong, China. E-mail: siewchienng@cuhk.edu.hk.

Jingwan Zhang, PhD, Department of Medicine and Therapeutics, The Chinese University of Hong Kong, 3/F, LiKS Medical Sciences Building, Prince of Wales Hospital, Shatin, Hong Kong, China. E-mail: wendyjwzhang@cuhk.edu.hk.

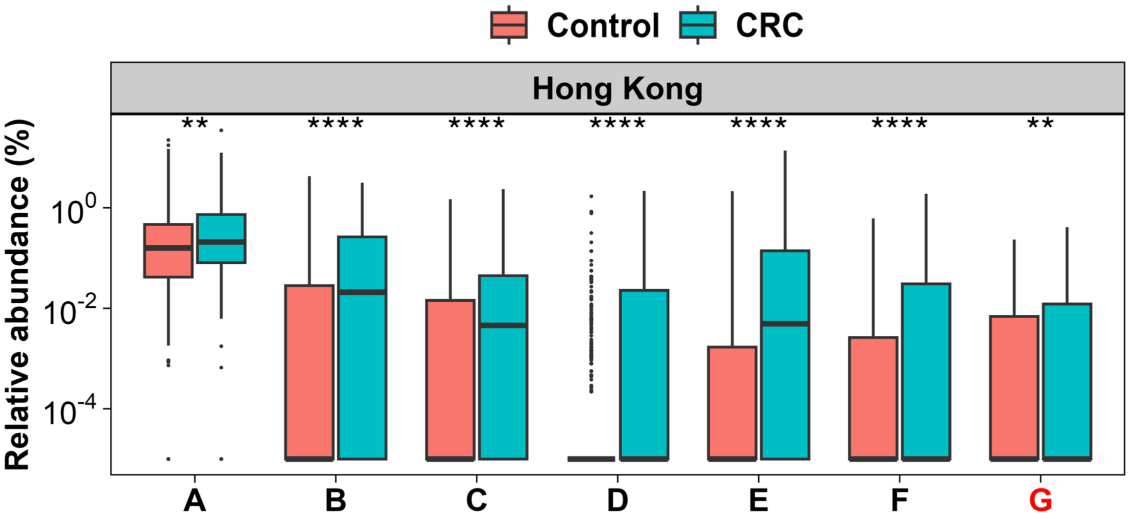

Fig. S1: Comparison of the relative abundance of 7 identified species between CRC patients and healthy controls in Hong Kong cohort. Statistical differences between groups assessed using the two-sided Mann-Whitney test. **P<0.001; ****P<0.0001. A-G represent the following species: *Ruthenibacterium lactatiformans*, *Alistipes shahii*, *Anaerotruncus colihominis*, *Peptostreptococcus stomatis*, *Parvimonas micra*, *Gemella morbillorum* and *Christensenella hongkongensis.*

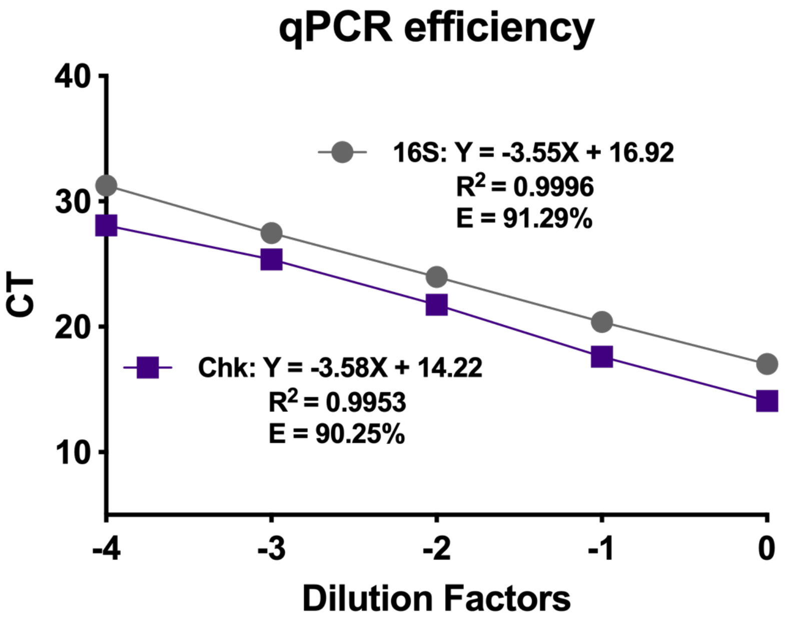

Fig. S2: qPCR amplification efficiency of *C. hongkongensis* and 16S rRNA primer sets. Standard curves were generated using 10-fold serial dilutions of template DNA to evaluate the amplification efficiency of the *C. hongkongensis* (purple squares) and 16S rRNA (gray circles) primer sets.

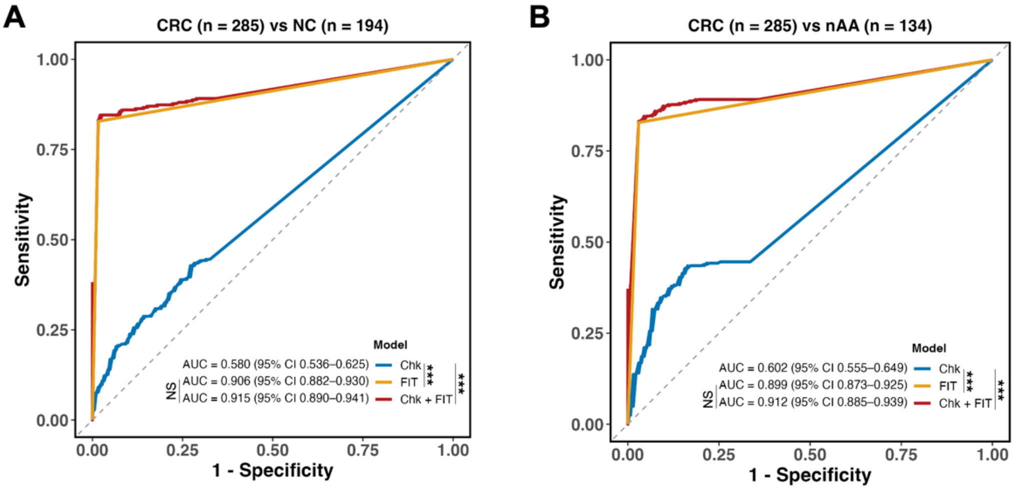

Fig. S3: Diagnostic performance of *C. hongkongensis* and FIT in single and combined testing in the mainland China cohort. Comparison the diagnostic performance **A** between CRC and NC groups; **B** between CRC and nAA groups. For each comparison, ROC curves represent the diagnostic accuracy of fecal *C. hongkongensis* (Chk; blue lines), FIT (orange lines), and their combined model (Chk + FIT; red lines). The area under the curve (AUC) values, 95% confidence intervals (CIs), and significance levels are shown within each panel. ***$P < 0.001$; NS, $P > 0.05$.

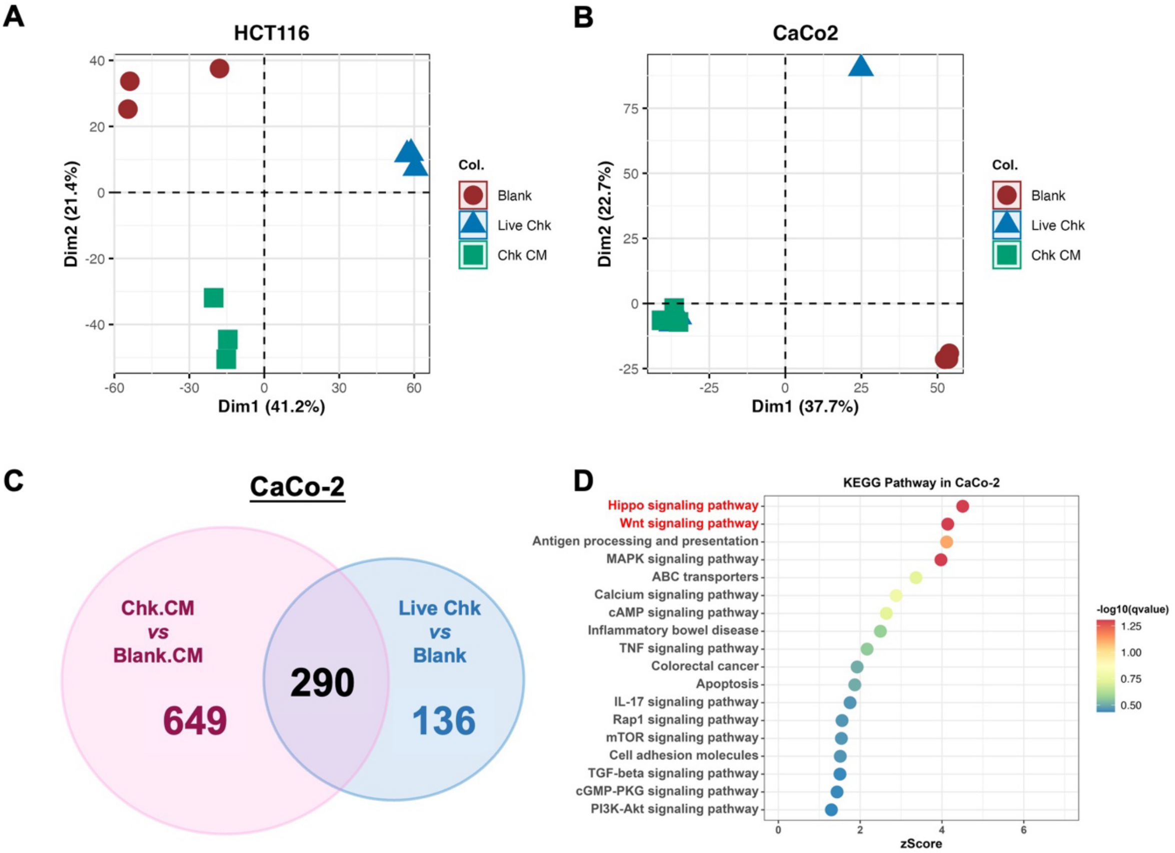

Fig. S4: Alteration of gene expression profiles in CRC cells after treatment with *C. hongkongensis* and its cultured medium. A and B PCA plots showing the distribution of genes across treatment groups in HCT116 (A) and CaCo-2 cells (B, n=3 in each group). **C** Venn diagram showing the intersecting differentially expressed genes (DEGs) between live *C. hongkongensis* and 5% Chk.CM treatments in CaCo-2 cells. **D** Bubble plot illustrating KEGG pathway enrichment of these intersecting DEGs in CaCo-2 cells.

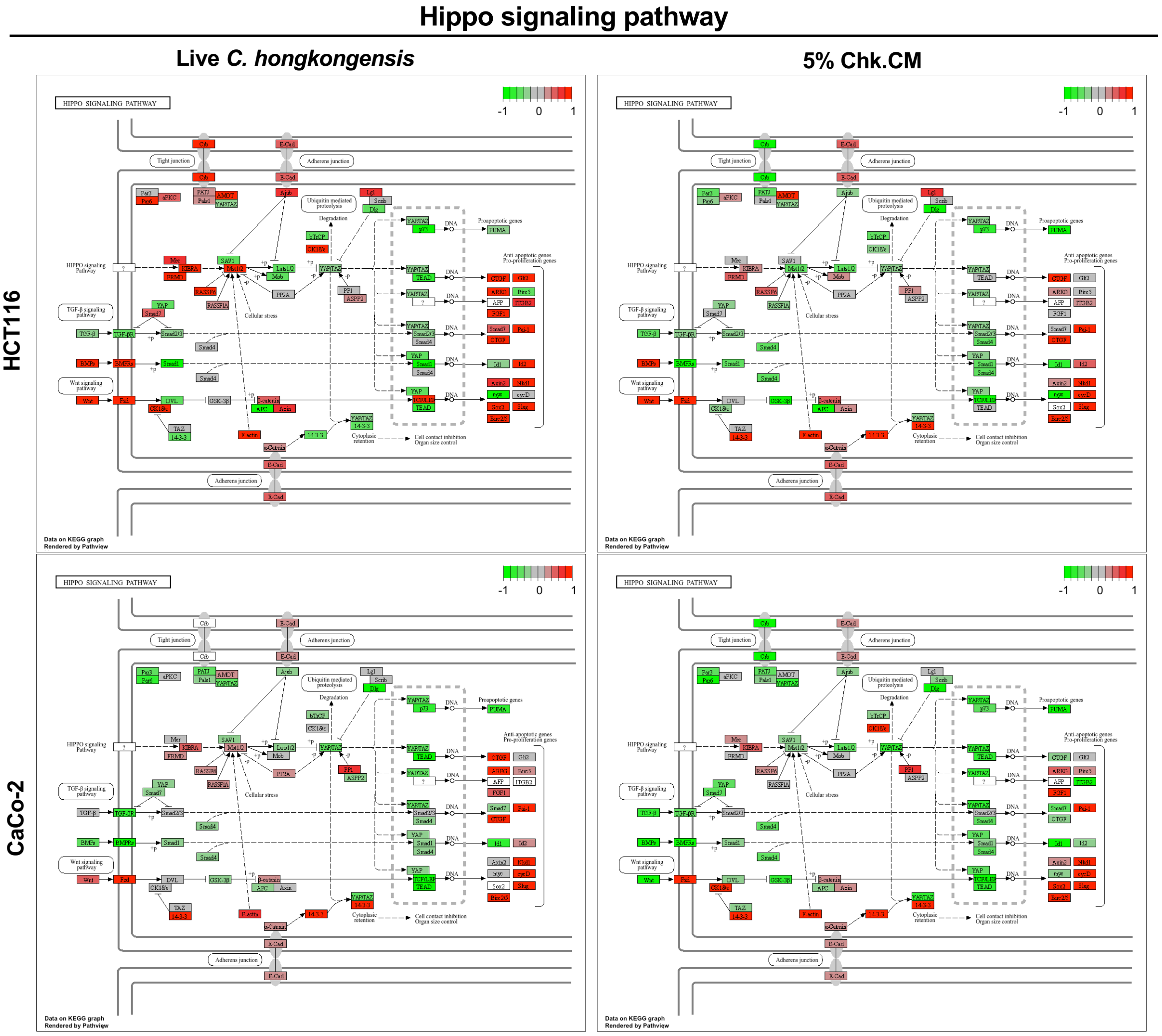

Fig. S5: Effects of live *C. hongkongensis* and its cultured medium on the Hippo signaling pathway in CRC cells. PathView analysis of RNA-seq data showing the regulation of the Hippo signaling pathway in HCT116 (top) and Caco-2 (bottom) cells after treatment with live *C. hongkongensis* (left) or 5% Chk.CM (right). Genes with increased expression are shown in red, while genes with decreased expression are shown in green. The intensity of the color represents the magnitude of the change, as indicated by the color scale bar (log2 fold-change).

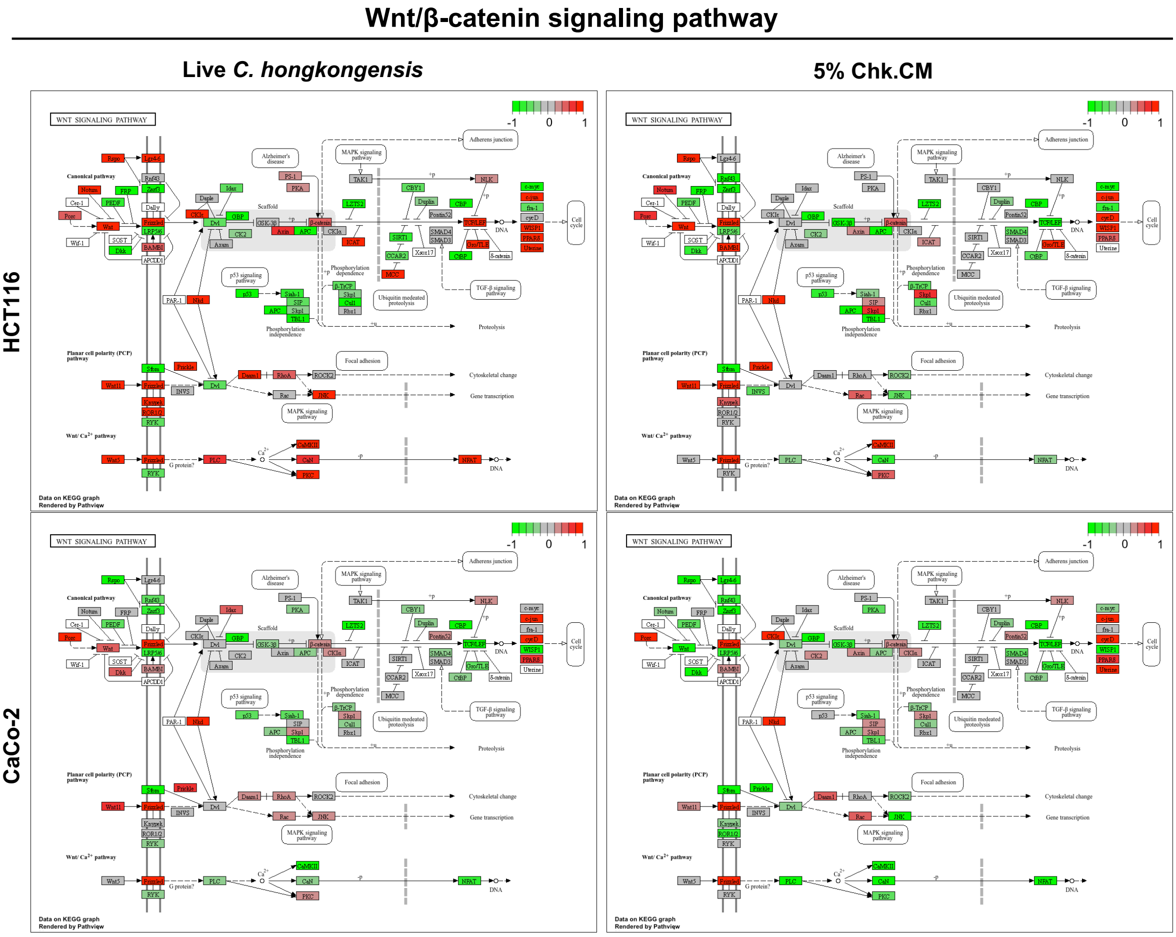

Fig. S6: Effects of live *C. hongkongensis* and its cultured medium on the Wnt signaling pathway in CRC cells. PathView analysis of RNA-seq data showing the regulation of the Wnt signaling pathway in HCT116 (top) and Caco-2 (bottom) cells after treatment with live *C. hongkongensis* (left) or 5% Chk.CM (right). Genes with increased expression are shown in red, while genes with decreased expression are shown in green. The intensity of the color represents the magnitude of the change, as indicated by the color scale bar (log2 fold-change).

Figure 5G and Figure 6D #1

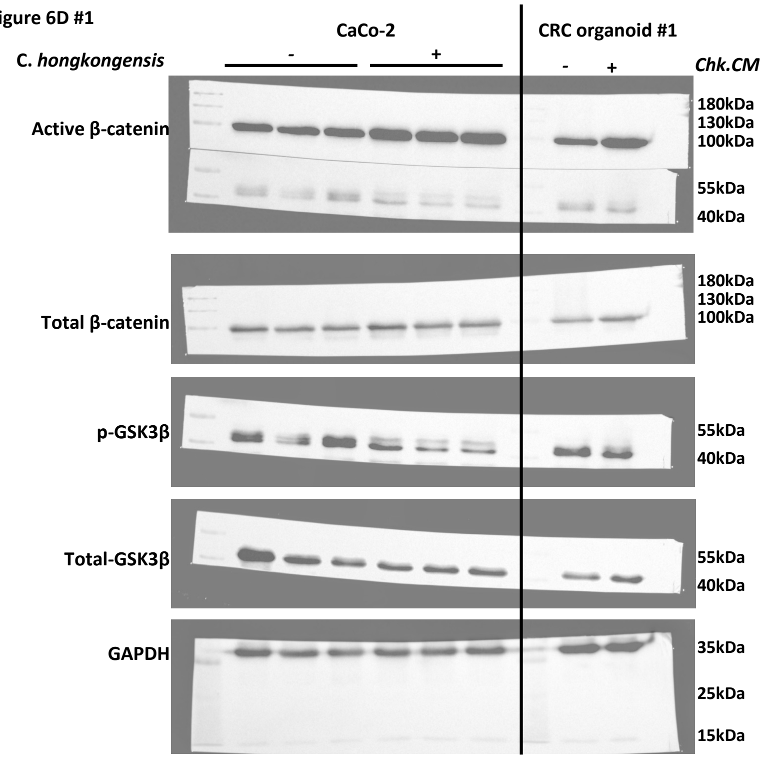

Figure 5H

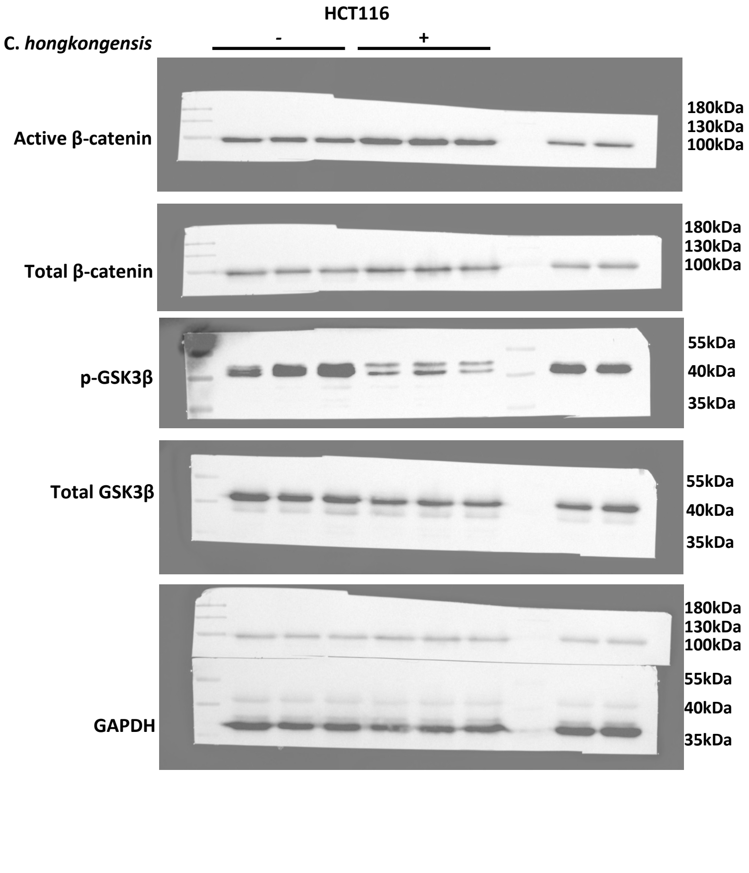

Figure 5J

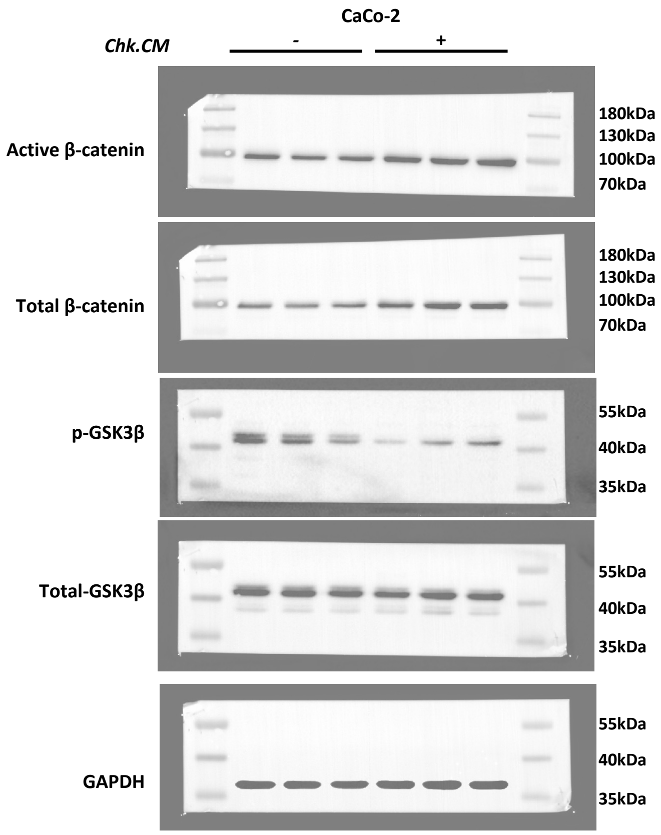

Figure 5K

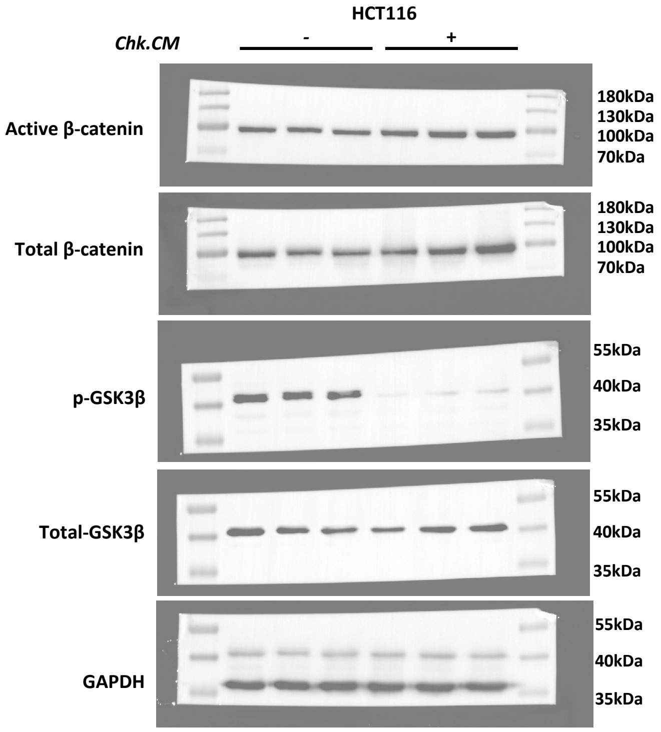

Figure 6D

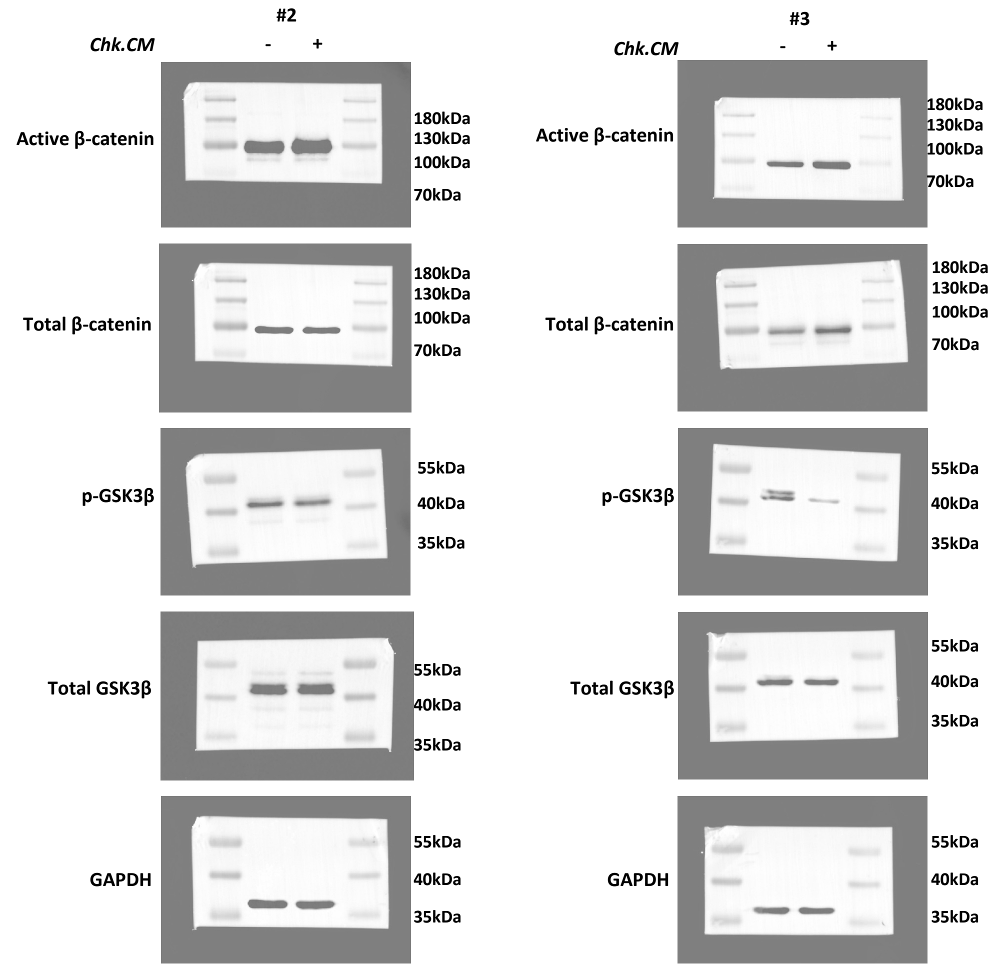
#2
#3
Chk.CM
-
+
Active β-catenin
Total β-catenin
p-GSK3β
Total GSK3β
GAPDH
180kDa
130kDa
100kDa
70kDa
55kDa
40kDa
35kDa

81
